# Evidence for Two Different Regulatory Mechanisms Linking Replication and Segregation of *Vibrio cholerae* Chromosome II

**DOI:** 10.1371/journal.pgen.1003579

**Published:** 2013-06-20

**Authors:** Tatiana Venkova-Canova, Jong Hwan Baek, Peter C. FitzGerald, Melanie Blokesch, Dhruba K. Chattoraj

**Affiliations:** 1 Laboratory of Biochemistry and Molecular Biology, Center for Cancer Research, National Cancer Institute, National Institutes of Health, Bethesda, Maryland, United States of America; 2 Genome Analysis Unit, Center for Cancer Research, National Cancer Institute, National Institutes of Health, Bethesda, Maryland, United States of America; 3 Global Health Institute, School of Life Sciences, École Polytechnique Fédérale de Lausanne, Lausanne, Switzerland; Institute of Molecular and Cell Biology (IMCB), A*STAR, Singapore

## Abstract

Understanding the mechanisms that coordinate replication initiation with subsequent segregation of chromosomes is an important biological problem. Here we report two replication-control mechanisms mediated by a chromosome segregation protein, ParB2, encoded by chromosome II of the model multichromosome bacterium, *Vibrio cholerae*. We find by the ChIP-chip assay that ParB2, a centromere binding protein, spreads beyond the centromere and covers a replication inhibitory site (a 39-mer). Unexpectedly, without nucleation at the centromere, ParB2 could also bind directly to a related 39-mer. The 39-mers are the strongest inhibitors of chromosome II replication and they mediate inhibition by binding the replication initiator protein. ParB2 thus appears to promote replication by out-competing initiator binding to the 39-mers using two mechanisms: spreading into one and direct binding to the other. We suggest that both these are novel mechanisms to coordinate replication initiation with segregation of chromosomes.

## Author Summary

Replication and segregation are the two main processes that maintain chromosomes in growing cells. In eukaryotes, the two processes are restricted to distinct phases of the cell cycle. In bacteria, segregation follows replication initiation with a modest lag. Influences of one process on the other have been postulated. The act of replication has been suggested to provide a motive force in chromosome segregation. Moreover, segregation proteins (ParA) have been found to interact with and control the replication initiator, DnaA. Here we show that in *V. cholerae* chromosome II, which is believed to have originated from a plasmid, a centromere binding protein (ParB) could control replication by two distinct mechanisms: spreading from a centromeric site into the replication-control region, and direct binding to the primary replication-control site, which has limited homology to the centromeric site. These studies establish that Par proteins can influence replication by at least three mechanisms. Homologous Par proteins participate in plasmid segregation but they are not known to influence plasmid replication. The expanded role of Par proteins appears likely to have been warranted to coordinate chromosomal replication and segregation with the cell cycle, which appears less of an issue in plasmid maintenance.

## Introduction

Studies in bacteria as well as in eukaryotes have shown that processes that maintain chromosomes, such as replication, recombination and repair, although able to occur independently of each other, often influence each other. Chromosome segregation is a major maintenance process but our knowledge of it in bacteria is relatively recent. This is because in well-studied bacteria such as *Escherichia coli*, genes dedicated to the segregation process have not been evident. Such genes were discovered in bacterial plasmids, called *parA* and *parB*. Subsequently, their homologs were found in a majority of sequenced bacterial chromosomes [1], [2]. Wherever tested, the chromosomal *parAB* genes were capable of conferring segregational stability on unstable plasmids bearing *parS* (“centromere” analogous) sites [3], [4], [5], [6], and made at least some contribution to chromosome segregation [7], [8]. In spite of limited study, it is becoming clear that chromosomal segregation systems can influence and be influenced by other chromosome maintenance processes.

In bacteria, replication and transcription have been proposed to provide motive force in chromosome segregation [9], [10], [11]. Coupled transcription-translation of membrane proteins is also thought to play an important role in chromosome segregation [12], [13]. One of the segregation proteins, ParB, can also spread and silence transcription of genes in its path [14], [15], [16]. The influence of segregation proteins in replication was suggested when ParB was found to load a condensin protein in the vicinity of the replication origin in *Bacillus subtilis* and in *Streptococcus pneumoniae* [17], [18], [19]. A more direct role was evident when ParA was found to influence the activity of the initiator DnaA in *B. subtilis* chromosome replication [20], [21] and in replication of *Vibrio cholerae* chromosome I (chrI) [22]. Recently, ParB encoded by *V. cholerae* chromosome II (chrII) was also found to influence chrII replication [23]. Here we report two distinct mechanisms for this ParB-mediated effect.

**Figure 1:**
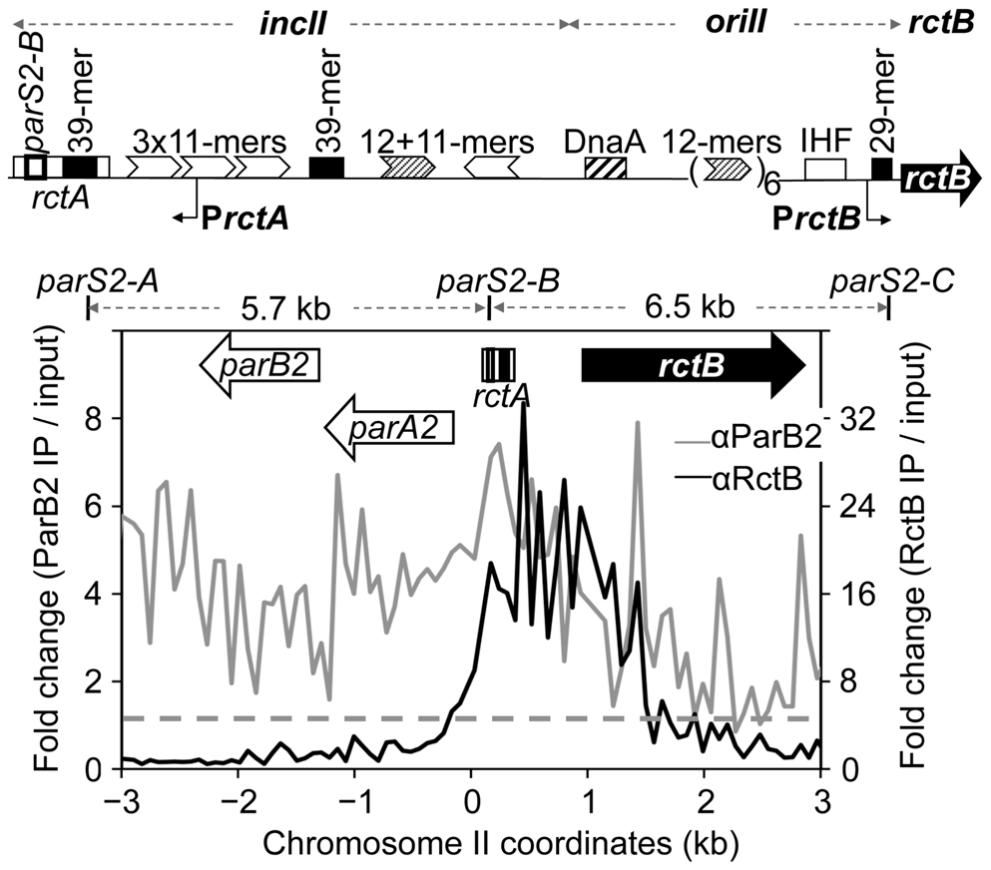
ParB2 spreads into the replication origin of *V. cholerae* chromosome II (chrII). The origin region has three functional units: *incII*, *oriII* and *rctB*. *incII* is required for controlling replication, *oriII* for initiating replication, and *rctB* for supplying the chrII-specific replication initiator protein, RctB. RctB binds to two kinds of site: the 11- and 12-mers (arrow heads) and 39-mers (black rectangles). The origin also has binding sites for DnaA and IHF, and the two promoters P*rctA* and P*rctB*. The *rctA* locus has a 39-mer and a binding site for ParB2, *parS2-B*. The bottom panel shows binding of ParB2 (grey profile) and RctB (black profile) in the origin and flanking regions, determined by ChIP-chip using specific antibodies (denoted as α) against the two proteins. The dashed line represents the average signal for ParB2 (1.1±1.1) over the entire genome. The corresponding value for RctB is 1.1±0.9. *parS2-A* and *parS2-C* are the nearest neighbors of *parS2-B*. The error bars here and elsewhere represent one standard deviation.

*V. cholerae* chrII replication is primarily controlled by its specific initiator protein, RctB [24], [25]. RctB binds to two kinds of site in the replication origin of chrII. One kind, the 11- or 12-mers, plays both essential and regulatory roles [26]. The other kind, two 39-mers and a 29-mer (a truncated 39-mer), plays only an inhibitory role in replication [26], [27]. One of the 39-mers is situated at a locus called *rctA*, at one end of the origin, and the other is more centrally located in the origin (Figure 1, top). The 29-mer is located in front of the *rctB* gene and is involved primarily in autorepression of the gene [27]. The *rctA* locus contains, in addition to a 39-mer, one of the ParB2 binding sites, *parS2-B* [28]. It has been reported recently that *parS2-B* alleviates some of the replication inhibitory activity of *rctA* in a ParB2-dependent fashion, but the mechanism is unknown [23]. Here we show that ParB2 spreads from *parS2-B* into the *rctA* 39-mer, and suggest that the spreading likely interferes with RctB binding to the 39-mer and thereby restrains the inhibitory activity of *rctA*. Unexpectedly, we also found ParB2 promotes replication by directly binding to the central 39-mer, without requiring spreading from *parS2-B*. We provide evidence that ParB2 competes with RctB for binding to the central 39-mer specifically and could thereby restrain its activity. In addition to revealing new ways by which a Par protein might influence replication, our results are significant in demonstrating that a segregation protein can bind specifically outside of centromeric sites.

## Results

### ParB2 can spread into the replication origin of chrII

The origin region of chrII comprises three functional units: A region required for controlling initiation (*incII*), a region minimally required for initiation (*oriII*) and a gene required for synthesizing the initiator protein (RctB) (Figure 1, top). The region covering the first two units, which consists mostly of sites for initiator binding, will be referred to as the origin. The *rctA* locus of *incII* exerts a strong inhibitory effect on chrII replication because of the 39-mer it contains [26]. The locus also has a site, *parS2-B*, for binding to the segregation protein ParB2, and the inhibitory effect of *rctA* is reduced in the presence of ParB2 [23]. Knowing that ParB proteins can spread out from their binding sites to neighboring sequences [14], we asked whether ParB2 spreading from *parS2-B* over the 39-mer might be a mechanism to control its inhibitory function. The extent of ParB2 spreading was tested by ChIP-chip analysis using antibody against ParB2 (Figure 1, bottom). Spreading was evident on either side of *parS2-B* (grey profile). In contrast, when the antibody was against RctB, the immunoprecipitated DNA in the origin region was restricted to where RctB has specific binding sites (black profile). These results suggest that ParB2 has the potential to modulate replication initiation activity by interfering with RctB binding.

### ParB2 can silence transcription in the origin of chrII

**Figure 2:**
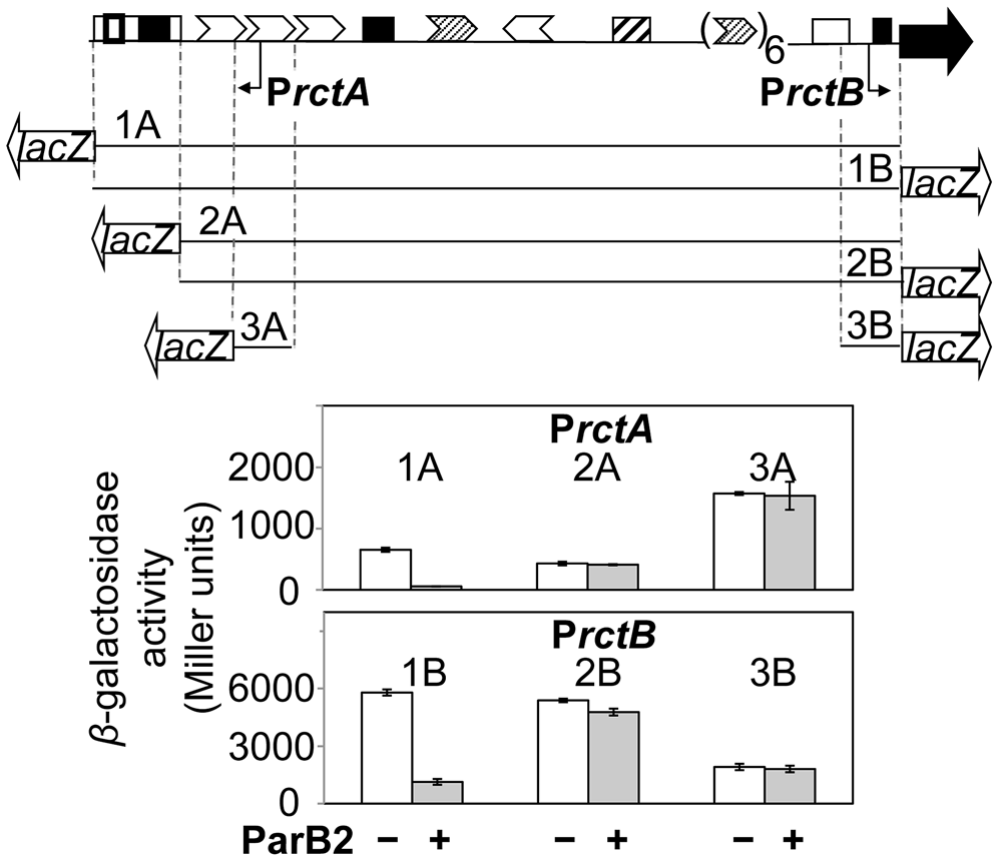
ParB2 can silence P*rctA* and P*rctB* in the presence of *parS2-B* in *E. coli*. The top map shows the origin region as in Figure 1. The lines below the map show the regions of the origin that were cloned upstream of a promoter-less *lacZ* gene and tested for promoter activity in *E. coli*. Fragments marked 1A–3A are present in pTVC122-124, respectively, and are orientated to record P*rctA* activity. The fragments marked 1B–3B are present in pTVC210-11 and pAS1, respectively, and are oppositely oriented to record P*rctB* activity. The white bars represent *β*-galactosidase activities obtained in the presence of an empty vector (pACYC184), while the grey bars indicate the activities in the presence of pTVC236 that supplied ParB2 at about 14-fold the physiological level. The copy number of *lacZ*-carrying plasmids was about 60 per cell, assuming there are four *oriC* copies in newborn *E. coli* cells in LB. The activities shown are mean values from three cultures inoculated with independent single colonies.

Spreading of proteins across genes can silence them [14]. For example, spreading into plasmid replication genes suitably close to a *parS* site can be lethal when selective pressure for plasmid retention is applied. By such an experimental test, we found that ParB2 can spread from *parS2-B* (Figure S1A). Spreading is also suggested by the formation of ParB2-GFP fluorescent foci in *parS2-B* carrying plasmids (Figure S1B). ParB2 could also silence two promoters, P*rctA* and P*rctB*, in the origin of chrII (Figure 1, top). P*rctA* is proximal to *parS2-B* whereas P*rctB* is located about 1 kb away at the other end of the origin. The activity of the promoters was assayed by fusing them to a promoter-less *lacZ* gene present in a multicopy plasmid in *E. coli* (Figure 2). The promoter fragments fused to *lacZ* carried either the entire origin including *parS2-B* (1A and 1B), or the origin lacking *parS2-B* (2A and 2B) or no additional DNA (3A and 3B). ParB2 was supplied constitutively *in trans* at about an order of magnitude higher than the physiological level (monitored by Western blotting; Figure S2), using P*trc* promoter without an intact *lac* repressor binding-site (*lacO*1), which makes the promoter unresponsive to IPTG. The presence of ParB2 reduced the activities of P*rctA* and P*rctB* significantly, only when the *parS2-B* site was present (Figure 2, 1A and 1B). These results suggest that ParB2 can spread over the entire origin in the presence of *parS2-B*, and does not have a significant effect on either promoter in the absence of *parS2-B*.

Since RctB has numerous binding sites in the origin, it appeared possible that RctB binding to them could counteract ParB2 spreading and reduce silencing of the promoters. This possibility was addressed by supplying RctB from an arabinose-inducible promoter, P_BAD_ [24], [26], [27], [29]. The induction of RctB alone (at about two-fold the physiological level) repressed P*rctA* marginally and P*rctB* about two fold (Figure S3, lanes 1 vs. 5). Silencing by ParB2 exceeded 90% for both the promoters (Figure S3, lanes 1 vs. 4). When RctB and ParB2 were supplied together, the repression of both the promoters was reduced about two fold compared to the level achieved with ParB2 alone (Figure S3, lanes 4 vs. 6; also inset). These results indicate that RctB can counteract the ParB2-mediated silencing.

In the results presented above (Figure 2 and Figure S3), the level of ParB2 was about 14-fold the level normally present in *V. cholerae* (Figure S2). When the concentration was reduced to about 10-fold, ParB2 could silence only the *parS2-B* proximal P*rctA*, but not the distal P*rctB* promoter (Figure S3, lanes 1 vs. 3). This reduced level of ParB2 was used in all subsequent experiments.

### ParB2 binds to the central 39-mer *in vivo*

**Figure 3:**
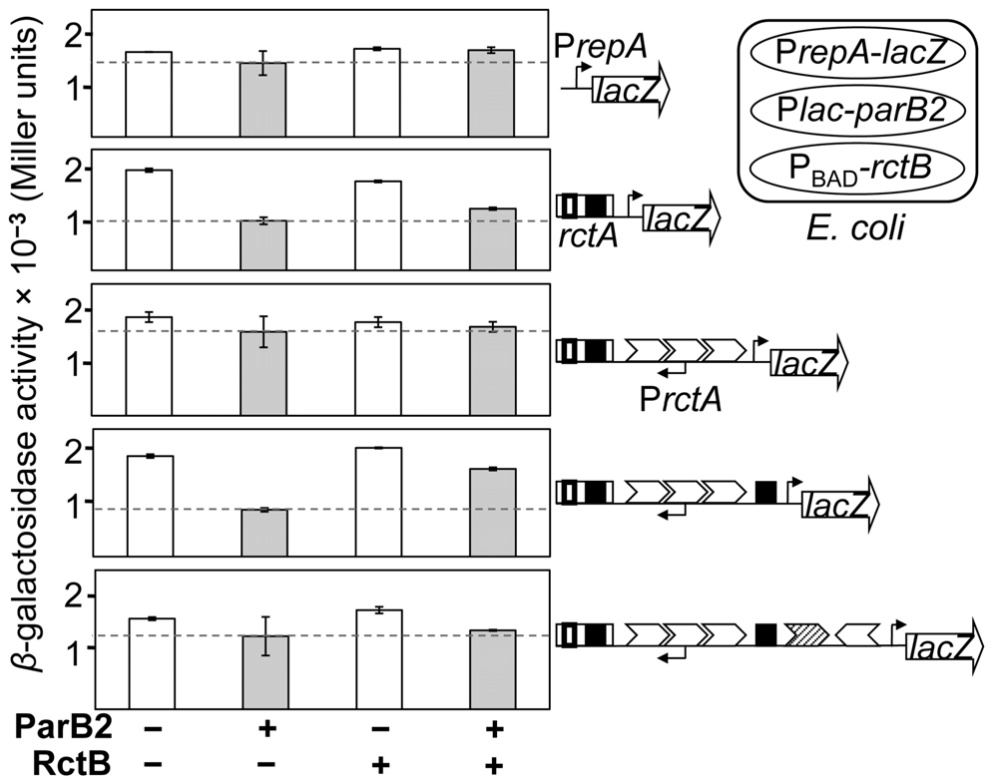
Silencing of a reporter promoter P*repA* by ParB2 without requiring *parS2-B*. A three-plasmid system was used in *E. coli* (cartoon in the top right corner) to monitor the span of ParB2-mediated silencing within *incII*. One plasmid carried various *incII* DNA fused to P*repA*, itself fused to a promoter-less *lacZ* gene, and the other two plasmids carried P*lac-parB2* (pTVC501) and Pbad-*rctB* (pTVC499), to supply ParB2 and RctB, respectively. The P*repA-lacZ* plasmids from the top were pTVC234, −239, −505, −504 and −509. The white and the grey bars represent *β*-galactosidase activities in the presence of uninduced (−) and induced (+) levels of ParB2. The induced level was about 10-fold the physiological level and the uninduced level was about an order of magnitude lower (Figure S2). The induced level (+) of RctB was about two-fold higher than the physiological level (Figure S1 of [27]). The uninduced level (−) of RctB was undetectable. The copy number of *lacZ*-carrying plasmids was about 60 per cell. The dotted lines represent *β*-galactosidase activities with induced levels of ParB2 alone, and are provided for comparison with activities under other conditions.

In order to determine how far ParB2 can spread beyond P*rctA*, progressively increasing lengths of *incII* were fused to a foreign reporter promoter, P*repA*, itself fused to *lacZ* [30] (Figure 3). In these experiments, in addition to a plasmid supplying ParB2, another plasmid was used to supply RctB. The two proteins were expressed from inducible promoters, P*lac* and P_BAD_, respectively (Figure 3, cartoon at the top right corner).

As expected, neither ParB2 nor RctB influenced the activity of P*repA* itself (Figure 3, top panel). In contrast, when *rctA* was present, ParB2 reduced the activity of P*repA* by two-fold (second panel). We believe this is due to ParB2 spreading from *parS2-B* into P*repA*, whose −35 box was only 167 bp away. RctB alone was ineffective, most likely because it does not spread, and its specific binding site, the *rctA* 39-mer, is well separated (by 85 bp) from the −35 box of P*repA*. Supplying RctB was marginally effective in relieving the silencing by ParB2. The next extension of the *incII* fragment included the 3x11-mers (third panel). Neither ParB2 nor RctB could silence the reporter promoter in this case. This result suggests that the spreading may not extend too far beyond P*rctA*. A further extension of the *incII* fragment by only 74 bp that included the central 39-mer, restored ParB2-mediated repression of the reporter promoter (fourth panel). This result was surprising since *parS2* sites were not found within *incII* [28]. The effect of ParB2 was significantly reduced when RctB was supplied, which is to be expected since RctB binds strongly to the central 39-mer [26]. This result suggests that ParB2 and RctB can compete for binding to the central 39-mer. The largest fragment (bottom panel) did not show a significant ParB2 effect on the reporter promoter, suggesting that ParB2 may not spread significantly from the central 39-mer. Together, the results suggest that under the conditions tested, ParB2 affects the origin primarily through interactions near *rctA* and the central 39-mer. The interaction near the central 39-mer suggests that ParB2 might bind there directly.

### ParB2 binds to the central 39-mer *in vitro*

**Figure 4:**
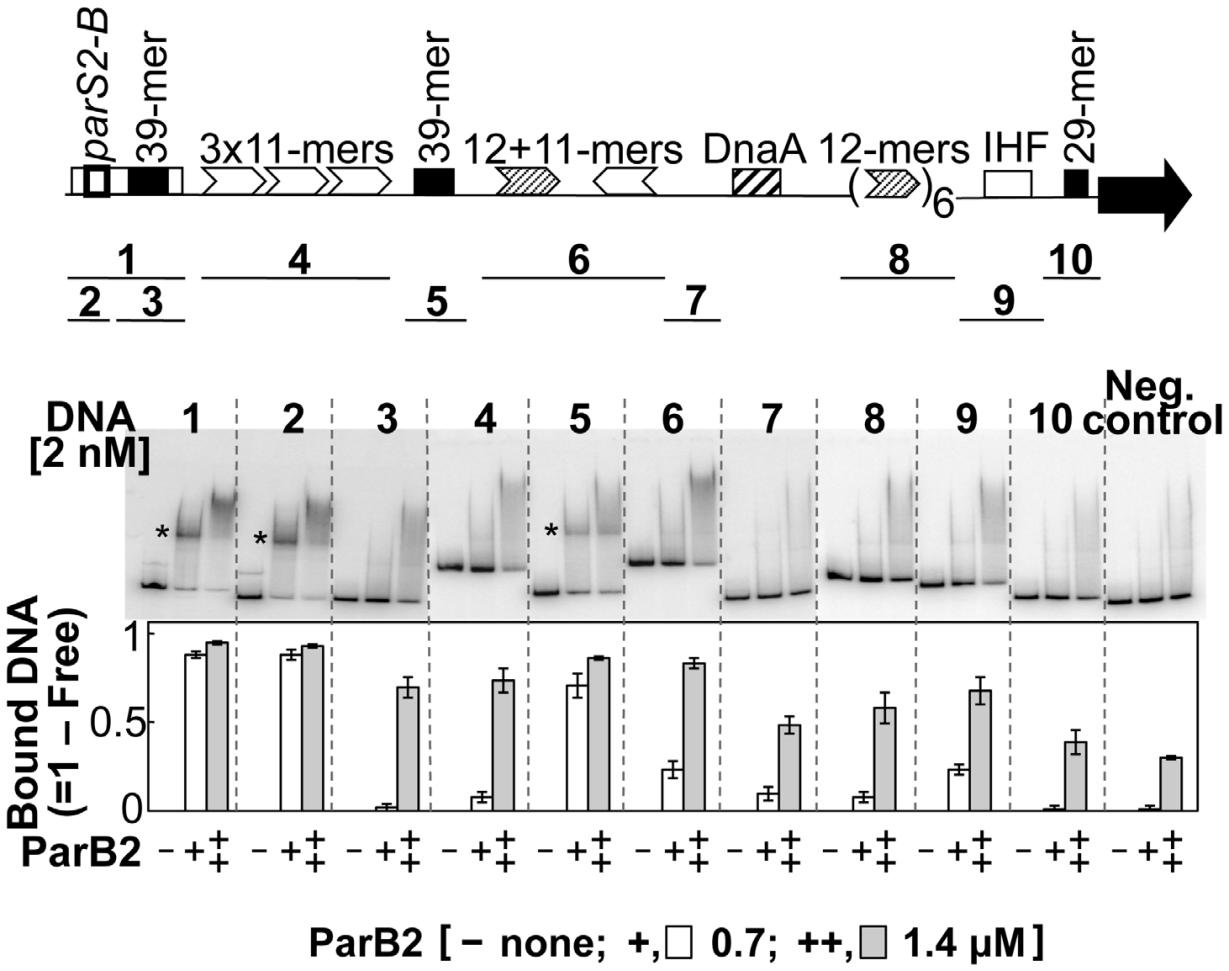
*parS2-B* and the central 39-mer are the only specific ParB2 binding sites in chrII origin. Binding was tested by EMSA using purified ParB2. The DNA fragments tested are indicated below the origin map. Fragments 1 to 10 were obtained by PCR from plasmids pTVC291, −526, −221, −248, −515, −270, −514, −228, −139 and −400, respectively. In addition to the origin sequences, all of the fragments had 100 bp of adjoining vector (pTVC243) sequences at each end. The fragment used as the negative control had only the identical vector sequences. Each fragment was run in three consecutive lanes with 0, 0.7 and 1.4 µM ParB2, marked by −, +, and ++, respectively, and the order was maintained for all the fragments. The stars mark the positions of specific ParB2 binding. The graph below shows the fraction of total DNA retarded by ParB2 at the indicated concentrations. The mean retarded fractions from three independent gels were plotted.

**Figure 5:**
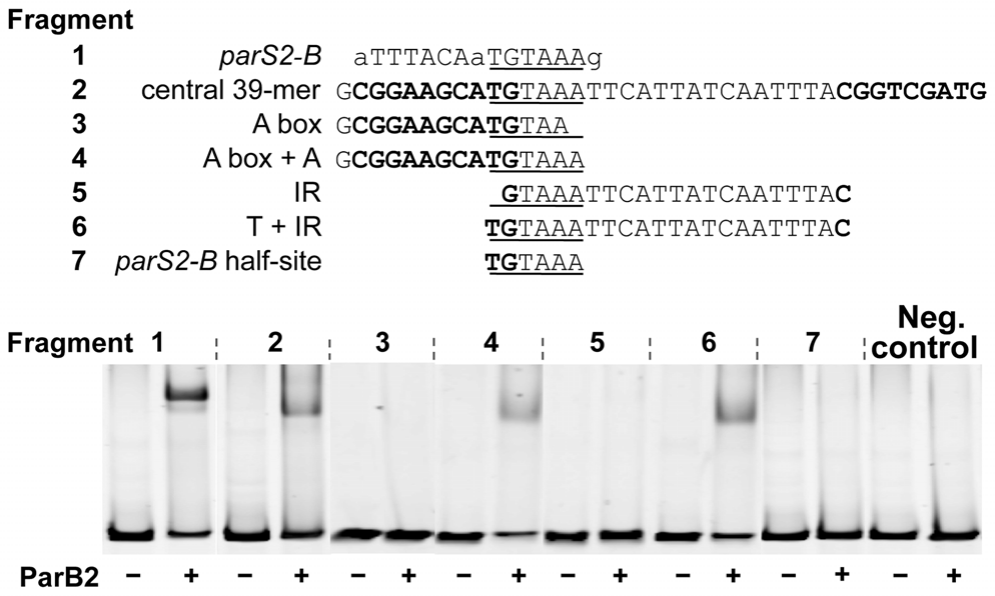
A ParB2 half-site is necessary but not sufficient for 39-mer binding. The experiments were done similarly to Figure 4, except for the insert sequences were as indicated. DNA fragments were used at 4 nM each and ParB2 at 1.4 µM (+). As in Figure 4, the insert sequences were flanked by BamHI (5′-GGATCC) and XhoI (5′-CTCGAG) sites of the vector, pTVC243. The fragments used in EMSA had additional 100 bp flanking sequences of the vector beyond those restriction sites. Fragments 1–7 were from plasmids pBJH200, pTVC222, pTVC132, pBJH216, pTVC120, pBJH221 and pBJH201, respectively.

The possibility of site-specific binding of ParB2 within the origin but outside of *parS2-B* was tested by EMSA. Several fragments covering the origin were used. Fragments 1 and 2, carrying the *parS2-B* site (positive controls), showed maximal ParB2 binding (Figure 4). The next significant binding was with the fragment containing the central 39-mer (fragment 5). This fragment contained natural flanking sequences of only 3 bp and 32 bp beyond the central 39-mer. The sequences (3+39+32) are exactly those that were added to the *incII* fragment of the third panel to generate the silencing-proficient fragment of the fourth panel (Figure 3). We found that the flanking sequences do not contribute to the central 39-mer binding (Figure S4, fragment #1). This result supports the inference from *in vivo* studies that ParB2 can directly bind to the central 39-mer without requiring *parS2-B*. Binding to the *rctA* 39-mer (fragment 3) was considerably weaker, possibly because the two 39-mers have several mismatches between them (Figure 1 of [26]; discussion related to Figure 5 below). The level of binding seen with the *rctA* 39-mer was comparable to the levels seen with fragments 4, 6, 8 and 9, and the level was marginally above that of the negative control that lacks any chrII sequences, suggesting that ParB2 has significant non-specific DNA binding activity.

The sequence requirement for specific binding of ParB2 to the central 39-mer was tested by variously mutating the sequence. The 39-mer has two conserved 9 bp direct repeats (called A and B boxes) flanking a 19 bp AT-rich spacer (Figure S4). The presence of both of the repeats and their proper phasing are important for RctB binding [26]. The AT richness of the spacer is also important but not the sequence per se. The *parS2* sites are AT-rich inverted repeats, only 15 bp long. Notably, the 39-mer spacer also contains an inverted repeat, which has some similarity to the consensus *parS2* site. However, the 39-mer spacer by itself was not sufficient for ParB2 binding; the presence of one of the direct repeats was necessary (Figure S4, fragments 4–6). Either of the direct repeats alone was also not sufficient (fragments 2–3). The inverted repeat feature could also be destroyed without compromising the binding efficiency (fragment 12). When the same fragments were tested for RctB binding, only the ones with the intact 39-mer and 10 bp deletion or addition (fragments 9–11) showed significant binding, as was also found earlier (data not shown; [27]). It appears that while both ParB2 and RctB bind to the 39-mer, the presence of one of the direct repeats is not obligatory for ParB2 binding.

Specific binding of ParB2 to the 39-mer was also verified by DNase I footprinting (Figure S5). Protection by ParB2 was conspicuous at the junction of the first direct repeat (A-box) and the AT-rich spacer. At this junction an intact *parS-2B* half site, 5′-TGTAAA, is present. This sequence is fully conserved in all 10 *parS2* sites that were competent in ParB2 binding [28]. In Figure S4, this half-site sequence was intact in all the binding positive fragments and mutant in all the fragments that failed to show specific binding. The half-site is also mutated to 5′-TTAAAC in the ParB2 binding-negative 39-mer in *rctA* (Figure 4, fragment #3). The half site thus appears to be necessary for 39-mer binding of ParB2. In further support of this inference, when we restored the original bases to some of the binding-negative 39-mer mutants to regenerate the half-site, binding proficiency was regained (Figure 5, fragments #3–6). Although necessary, the half site was not sufficient for binding ParB2 (fragment #7). We conclude that extension of the half site either to the left or right is necessary. This is not surprising since the affinity drops by orders of magnitude when one half of a dyad symmetric site is mutated [31], [32]. The minimal size of the extensions needed to regain binding activity of ParB2 remains to be determined.

### ParB2 and RctB compete for binding to the central 39-mer *in vitro*

**Figure 6:**
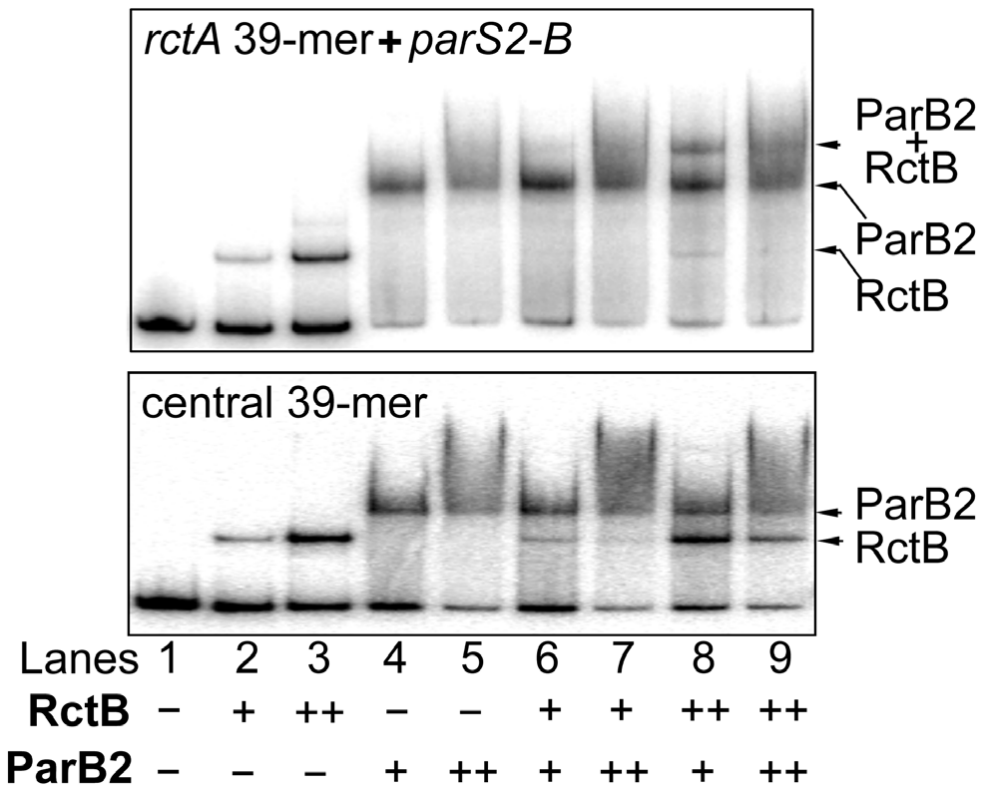
ParB2 and RctB bind simultaneously to *rctA*, but competitively to the central 39-mer. The fragment in the top panel contained the entire *rctA*, which has *parS2-B* and a 39-mer for binding ParB2 and RctB, respectively. The fragment in the bottom panel contained only the 39-mer central to *incII*. The fragments were PCR amplified from pTVC291 and pTVC222, respectively. (The *rctA* fragment is identical to the fragment 1 of Figure 4 but the 39-mer fragment does not have the natural flanks of the fragment 5 of Figure 4). The fragments (2 nM each) were subjected to EMSA with purified RctB and ParB2, each at two concentrations: 3 nM (+) and 30 nM (++) for RctB, and 0.7 µM (+) and 1.4 µM (++) for ParB2. Arrows indicate the bands representing single or simultaneous binding.

ParB2 and RctB can bind to *rctA* simultaneously [23] (Figure 6, top panel). This is not surprising since there are 34 bp of spacer sequence between the binding sites of the two proteins, and that ParB2 does not spread in vitro. The sites also remain functional when isolated from each other [33]. On the other hand, at the central 39-mer, the binding sites for the two proteins appeared to be largely overlapping, suggesting that they could not bind simultaneously. This was indeed the result (Figure 6, bottom panel). Even at the higher protein concentrations (++), no new discrete species representative of dual binding was detected. The results indicate that ParB2 and RctB compete for binding to 39-mer, unlike the simultaneous binding that can occur on *rctA*.

### ParB2 can control *oriII* copy number without requiring *parS2-B*

**Figure 7:**
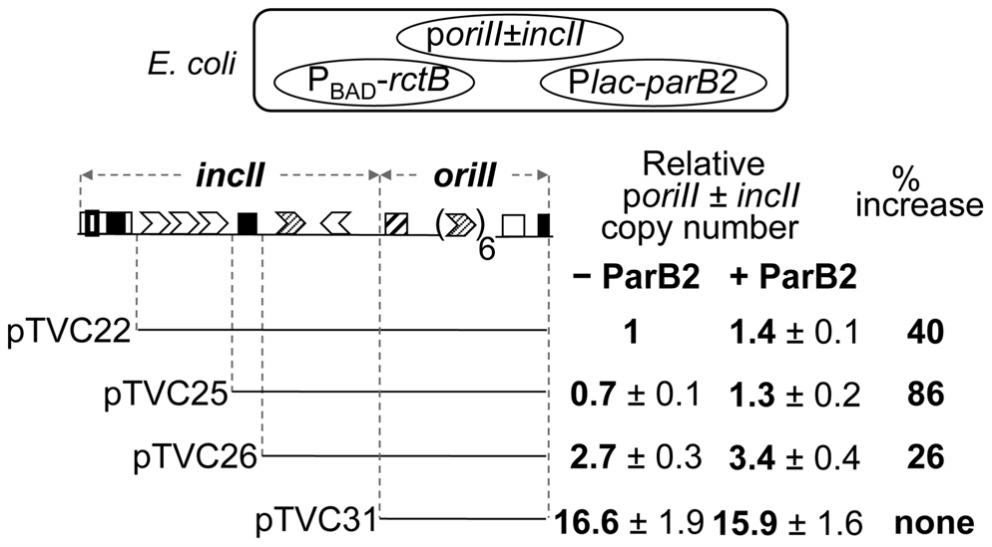
Increase in copy number of *oriII* plasmids by ParB2 in the absence of *parS2-B* in *E. coli*. The copy number was measured in *E. coli* by supplying RctB from Pbad-*rctB* present in pTVC499, and ParB2 from P*lac-parB2* present in pTVC501 (cartoon on the top). Lines below the origin map indicate the extent of origin DNA present in different plasmids. The copy-numbers were from cultures containing 0.002% arabinose that supplied a near-physiological level of RctB, and either no IPTG (− ParB2) or 100 µM IPTG (+ ParB2) that supplied ParB2 at about 10-fold the physiological level (Figure S2). The copy numbers are the mean values from three cultures inoculated with independent single colonies. The copy number 1 corresponds to four copies of *oriII* is per cell. % increase (last column) = 100×[Copy # (+ParB2)−Copy # (−ParB2)]/Copy # (−ParB2).

We previously showed that the central 39-mer is the most potent replication inhibitory site in *incII* and it functions through RctB binding [26]. If ParB2 competes with RctB for binding to the central 39-mer, this competition appeared likely to influence *oriII* activity without requiring the *parS2-B* site. This prediction was tested by determining the copy number of *oriII*-driven plasmids (Figure 7). The copy number of *oriII* plasmids depends on the extent of the *incII* sequences present [26]. Although the 39-mers are always inhibitory to replication, the 11- and 12-mers can either promote or inhibit replication depending upon whether the 39-mers are present or not. In the present experiments also, the *oriII* plasmid copy number first decreased and then increased with increasing deletion of *incII* (Figure 7, − ParB2 column). When ParB2 was additionally present, the copy number increased significantly in the first two 39-mer-carrying plasmids, the increase being maximal for the plasmid with the lowest copy number (pTVC25). In this plasmid, we suggest that the 39-mer was unencumbered by the 3x11-mers, and was maximally available for binding to ParB2. Together, these results indicate that ParB2 has the potential to facilitate chrII replication by restraining the inhibitory activity of the *incII* sequences, and can do so whether *parS2-B* is present or not.

### Roadblock of ParB2 spreading compromises the replication stimulatory activity

**Figure 8:**
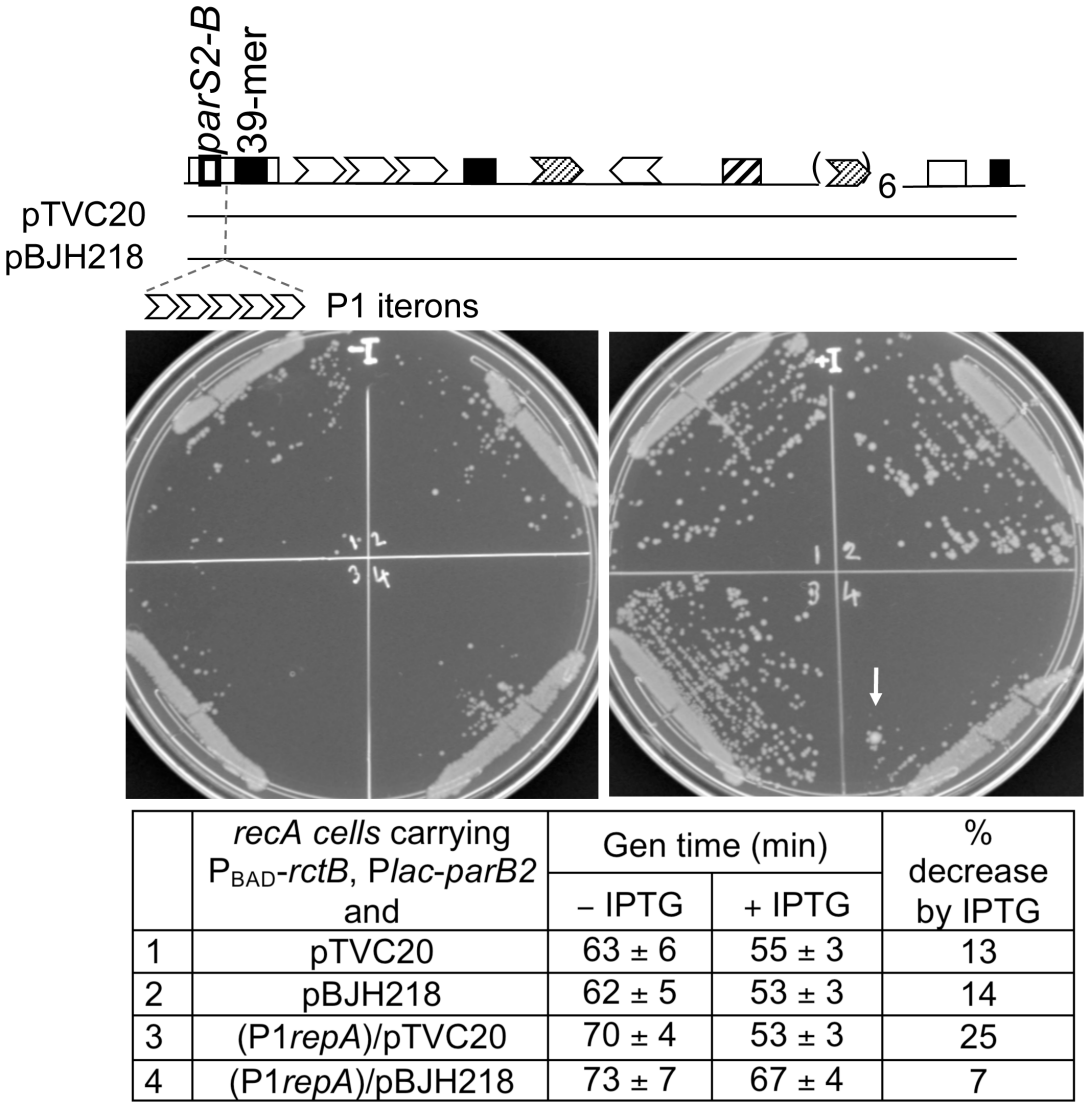
Effect of a roadblock on ParB2-mediated stimulation of *oriII* activity in ***E. coli***. Two *oriII* plasmids, pTVC20 and pBJH218, isogenic except for about 100 bp extra sequences carrying an array of five P1 RepA binding sites (iterons) in the latter, were used to transform BR8706 (*recA* minus) cells without (#1, #2) and with (#3, #4) an integrated λ prophage (λDKC311). The prophage supplied P1 RepA constitutively. The recipient cells also had pTVC499 and pTC501 to supply RctB and ParB2, respectively, as in Figure 7. Cultures from single colonies were grown to OD_600_≅0.1 with (+) or without (−) 100 µM IPTG. One µl of the cultures was streaked onto respective plates with and without IPTG for comparison of colony sizes. For determining generation times, the cultures were diluted 50× and grown to early log phase. The generation times are from five different cultures, and they decreased in the presence of IPTG in all cases, but less so under the condition RepA was expected to bind to P1 iterons and create roadblock (Table row #4). Note that in the IPTG carrying plate (+I), the growth was least robust for #4 compared to the other three. In that sector, a larger colony forming revertant is also conspicuous (+I plate; arrow). In these experiments, ParB2 was supplied at about 10× the physiological level, and *oriII* number was approximately one per cell.

If ParB2 spreading is one of the mechanisms by which the protein stimulates chrII replication, it might be possible to restrain this activity by placing a roadblock in the path of spreading. To this end, we inserted an array of five P1 RepA binding sites (iterons) between *parS2-B* and the 39-mer in *rctA* (Figure 8). The effectiveness of the roadblock in preventing the spreading of P1 ParB protein was demonstrated earlier [34]. Comparison of the top two rows of the Table in Figure 8 shows that in the absence of RepA (that is in the absence of a roadblock), ParB2 was equally efficient in promoting cell growth that depended on the functioning of *oriII* plasmids. In other words, the P1 iterons in pBJH218 did not compromise ParB2 spreading in the absence of the roadblock. The same two plasmid-carrying cells behaved differently in the presence of RepA (the last two rows). Upon induction of ParB2 production by IPTG, cell growth improved more in the case of pTVC20 than in the case of pBJH218. In other words, ParB2 effect was compromised under the condition the roadblock was expected to be effective. These results are consistent with ParB2 spreading as a mechanism for stimulating chrII replication initiation. Note that some increase of growth rate was seen even when ParB spreading was inhibited by a roadblock (generation time decreased 7% for cells in row #4). This result is not surprising because ParB2 can bind to the central 39-mer without requiring spreading from *parS2-B*. Overall, the ParB2 effects were modest, which is to be expected because of the existence of multiple controls on chrII replication.

### The central 39-mer is not a centromere

In chromosome and plasmid segregation, ParB proteins serve to couple centromeres to ParA proteins (NTPases) and modulate the NTPase activity that is believed to provide the movement required for segregation [8]. The binding ParB2 to a 39-mer raises the possibility of an inherent centromeric function of the site. This was tested by cloning the central 39-mer into a miniF plasmid, which is unstable due to deletion of its own segregation genes [35]. The stability of the miniF plasmid improved with the inclusion of the *parS2-B* site but not with the 39-mer, when ParA and ParB proteins were supplied in trans (Figure S6). This result suggests that ParB2 binding to *parS2-B* and the central 39-mer is different in an important respect.

## Discussion

**Figure 9:**
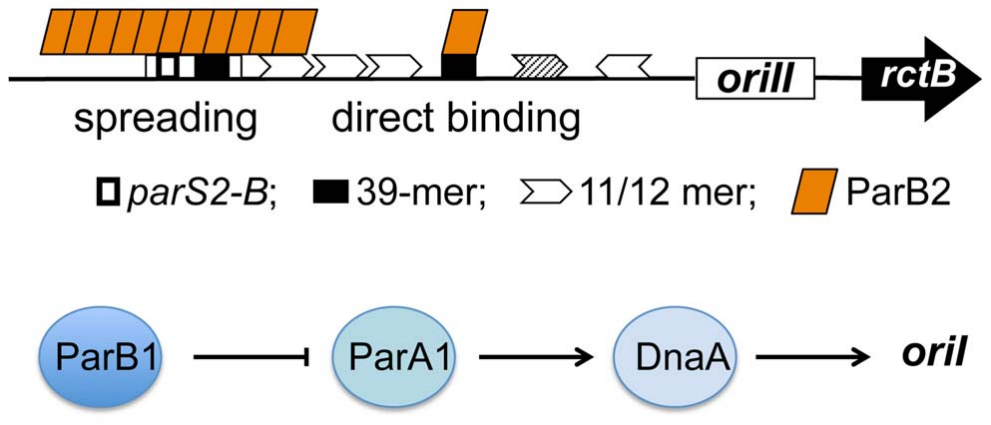
Model of activation of *V. cholerae* chromosome replication by Par proteins using multiple mechanisms. The upper diagram shows ParB2 interaction with the chrII origin by two mechanisms: spreading from the centromeric site *parS2-B* into a neighboring 39-mer and direct binding to another 39-mer. We suggest that these interactions promote chrII replication by out-competing initiator binding to the 39-mers. The lower diagram shows activation of chrI replication by ParA1 interaction with DnaA, which is negatively controlled by ParB1. For both chromosomes, the Par proteins target the most powerful regulators, by DNA-protein interactions for chrII and by protein-protein interactions for chrI.

Our knowledge of interactions between the processes of replication and segregation of chromosomes in bacteria is rather recent and limited. An interaction between the universal bacterial replication initiator, DnaA, and a segregation protein, ParA, was recently discovered in *B. subtilis* [20], [21], [22], [36] and later in *V. cholerae* where it was specific for chromosome I [22]. In both cases, replication initiation was modulated by ParA. A partner segregation protein, ParB, was found to affect replication of chromosome II (chrII) in *V. cholerae*. This parB, called ParB2, somehow promoted replication by binding to one of its cognate centromeric sites (*parS2-B*) [23]. Here we show that ParB2 spreads out of this centromeric site into the replication origin of chrII, and suggest that this spreading is a mechanism by which ParB2 promotes replication. We also report an additional mechanism by which ParB2 can promote chrII replication: direct binding to a replication inhibitory site in the origin. To our knowledge, the present results provide the first examples of a replication activation mechanism that is mediated by spreading of the activator from a distant site, and by the specific binding of a segregation protein outside of the centromere. These studies have revealed at least three ways by which segregation proteins can influence replication (Figure 9).

### ParB2 interferes with RctB binding

The spreading of ParB2 from a centromeric site into the origin of chrII was evident from *in vivo* cross-linking experiments (Figures 1,S7), from silencing of promoters within the origin (Figure 2) and from the reduction of reporter promoter activity when natural initiator (RctB) binding sites were present between the centromeric site and the promoter (Figure 3, third panel; Figure S3, insets). This latter result indicates that RctB binding could create a natural roadblock to ParB2 spreading. The fact that the span of silencing lengthened with increased ParB2 concentration (Figure S3) also supports the idea that the underlying mechanism involves spreading along the DNA. Finally, the results of placing an artificial roadblock were also consistent with the spreading mechanism (Figure 8). When a powerful replication inhibitory site (the *rctA* 39-mer) was present within the span of spreading, growth of cells dependent upon the functioning of the chrII origin improved. It was also reported earlier that ParB2 could increase replication of chrII origin carrying plasmids when they included the adjacent *rctA* region [23]. This increase was shown to be dependent on the presence of *parS2-B*. Together with the finding that ParB2 does not directly bind to the *rctA* 39-mer (Figure 4), and cannot spread from its binding site in the central 39-mer as discussed below (Figures 3 (last panel), S1A, S1B), the simplest explanation of these results is that by spreading from *parS2-B*, ParB2 compromises the inhibitory activity of the *rctA* 39-mer by interfering with RctB binding.

ParB2 was also found to reduce the activity of another potent replication inhibitor (the central 39-mer) without requiring the centromeric site and spreading (Figure 3, S1). The latter effect appears to be due to direct binding of ParB2 to the central 39-mer. This mode of ParB2 interaction with the 39-mer most likely also causes interference with RctB binding to this site (Figure 6). Since the 39-mers are the two sites most inhibitory to chrII replication and their activities are mediated through RctB binding, the reduction in binding suffices to explain how ParB2 could promote replication of chrII (Figures 7,8). Interference with binding of regulatory proteins to DNA by the spreading of a competing protein along DNA has also been invoked to explain transcriptional silencing, inhibition of DNA methylation and of DNA gyrase binding, and resistance to DNase I cleavage [37], [38], [39], [40].

### ParB2 does not interact with RctB directly

Although the only model we have entertained so far to explain the ParB2 effect is interference with specific binding of RctB, we have also tested whether ParB2 and RctB could interact directly. This possibility was suggested by the finding that ParA influences replication by protein-protein interaction rather than DNA-protein interaction [20], [21], [22], [36]. However, ParB2 did not show any detectable interaction with RctB, as was also reported earlier (Figure S8) [23].

### Influence of ParA2 on binding and spreading of ParB2

ParA participates in a number of processes involving ParB [8]. Here, we asked whether binding of ParB2 to the central 39-mer is also influenced by ParA2. The binding was assayed indirectly by fusing a foreign promoter close enough to the 39-mer that ParB2 binding to the site could interfere with the promoter activity. The promoter activity did not change significantly upon supply of ParA2 (Figure S9; data with P*repA*). This suggests ParB2 binding to the 39-mer is not influenced by ParA2. We did find a minor influence of ParA2 on P*rctA* silencing by ParB2 spreading, the basis of which was not explored. In the case of P1 plasmid and *B. subtilis* chromosome, no ParA effect on ParB spreading was evident [14], [41].

### *E. coli* as a host to study chromosome II

Whereas deletion of *parS2-B* in *V. cholerae* was easily tolerated (Figure S7; [23]), deletion of the *parB2* gene was essentially lethal [42]. In the absence of ParB2, chrII loss is evident at every cell division that causes a severe growth defect. We were therefore unable to test conveniently the role of ParB2 on replication of chrII in the native host. On the other hand, there was no obvious effect of ParB2 on the growth of *E. coli*, in which we did most of our experiments. The validity of extrapolating the observations in *E. coli* to the native host appears warranted by the observation that ParB2 effects were seen in the context of the entire origin (Figure 8; [23]), and by the finding that some of the inferences from the *E. coli* results were valid when tested *in vitro* (Figure 6). In the past, wherever chrII replication control was studied in both *E. coli* and *V. cholerae*, the results agreed [25], [26], [28], [29]. Nonetheless, the ParB2 concentration required for spreading to proceed just over *rctA* was an order of magnitude higher than that is normally present in the native host. The reason for this discrepancy is not understood but a possibility is that ParB2 when supplied from a *trans* source is much less effective. A discrepancy in the amount of protein required from a *cis* vs. *trans* source has been noted in the case *V. cholerae* ParA1 [22] and ParA of *Pseudomonas aeruginosa* [43]. The production of one of the Par proteins without its partner could also have altered the protein activity and stability. The importance of maintaining the stoichiometry of Par proteins has also been indicated in studies of *B. subtilis* [20], [44]. Another possibility is that higher protein concentration may be required to bind to a single *parS* site, as we have used here, than when there exists neighboring sites, as in the native host, that might allow cooperative binding.

### Physiological relevance of ParB2 spreading

We show that in wild type *V. cholerae* cells ParB2 can bind and spread over the entire origin (Figure 1). We detected considerable ParB2 spreading, even with the deletion of the origin proximal centromeric site (*parS2-B*) (Figure S7). Most likely, this spreading originates from the neighboring *parS2-A* site 5.7 kb away (Figure 1). The spreading could add an additional layer of control over P*rctA* by silencing the promoter, which is independently repressed by RctB (Figure S3) [24], [45]. The P*rctA* activity in turn controls RctB binding to the *rctA* 39-mer [29]. The multiple feedback loops that operate to control the initiation of replication from the origin of chrII appear securely interlocked with the specific segregation system of this chromosome. The presence of multiple layers of control could compensate for a deficiency in any one of the regulators, and help in homeostasis of origin copy number.

The finding that ParB2 could spread over the entire origin might suggest that it could be a mechanism to promote chromosome segregation. It might increase the effective size of the kinetochore, which might facilitate its interaction with ParA, the essential partner of ParB in chromosome segregation. However, this role has yet to be established [16], [34], [46].

### Evolutionary considerations

ParB proteins of plasmids are known to be plasmid-specific and to bind to their cognate sites [47]. This helps to avoid segregation-mediated incompatibility if different plasmids happen to be present in the same host. By the same token, in multichromosome bacteria, the segregation systems should be chromosome-specific. Such is clearly the case in *Burkholderia cenocepacia* [5] and in *V. cholerae* [4], [48]. The same ParB protein has been found to bind to variant *parS* sites but the sites are believed to be descendants of a common ancestor [49]. In this context, it is noteworthy that although the central 39-mer is largely non-homologous to *parS2-B*, the region of the 39-mer crucial for ParB2 binding shares six bp of perfect identity with *parS2-B* (Figures 5,S4), suggesting the possibility of an evolutionary link between the sites here also.

Chromosome segregation begins soon after replication initiation, thereby compressing the total time for the completion of these two processes. Their close coordination also allows segregation to proceed in a more orderly fashion than if the substrate for segregation were a pair of completed and entangled sister chromosomes. Here, we have described interactions that might assist in coordinating replication initiation and segregation. In *V. cholerae*, following replication initiation, the majority of the RctB binding sites (11- and 12-mers) stay hemi-methylated and are unable to bind the initiator [50]. This stage of the cell cycle should favor spread of ParB2 into the origin (Figure S7), which is likely to favor origin segregation and at the same time discourage premature reinitiation. Spreading of ParB2 towards the origin is apparently prevented later in the cell cycle when the origin is remethylated, allowing RctB binding to 11- and 12-mers that eventually leads to initiation (Figure 3, 3^rd^ panel). At these latter stages, when spreading is blocked, direct binding of ParB2 to the central 39-mer should favor initiation. Thus, depending upon the stage of the cell cycle, ParB2 appears to play opposite roles in controlling chrII replication, but in such a way as to promote the orderly sequence of chromosome replication followed by segregation. In the case of plasmids, which can complete replication in a tiny fraction of the cell division cycle, such coordination is neither necessary nor evident. We suggest that the acquisition of interactions such as we describe are a feature of the putative adaptation of an acquired plasmid to permanent residency as a second chromosome.

## Materials and Methods

### Strains and plasmids

*V. cholerae* and *E. coli* strains, and plasmids used in this study are listed in Table S1. ChrII fragments were amplified from N16961 (CVC209) DNA by PCR using Phusion High-Fidelity polymerase (NEB, Beverly, MA). The sequences of primers used for PCR are shown in Table S2. For cloning sequences up to 100 bp, complementary oligonucleotides (IDT, Skokie, IL) were used after annealing the two [29]. The exact chrII coordinates of each cloned fragment are given in Table S1.

### *β*-galactosidase assay

This was done in L broth cultures of *E. coli* strain BR8706 at OD_600_ between 0.4–0.5, as described [24]. To account for any effect that ParB2 might have on the replication of the *lacZ*-reporter plasmid, *β*-galactosidase activities were normalized for the plasmid copy number in all cases. The copy number variation was small; one standard deviation was within 20% of the mean. The copy numbers were measured (see below) from aliquots of the same cultures that were used for *β*-galactosidase measurements. Some of the cultures were simultaneously monitored for ParB2 amounts by Western blotting (Figure S2). Note that +/− ParB2 refer to cells carrying pTVC501 (that carries *parB2* under IPTG control) with and without IPTG induction, respectively. In Figure 2 and Figure S3 (lanes 4,6), + refers to cells carrying pTVC236, which supplies ParB2 from a constitutive promoter and − refers to cells carrying the empty vector, pACYC184.

### Plasmid copy number measurement

The copy number of *lacZ*-carrying plasmids (Figures 2,3, S3, S9) were measured exactly as described [32]. Briefly, different experimental cultures were grown to log phase and mixed with separately grown cells carrying pNEB193 before plasmid isolation. The latter plasmid helped to account for plasmid loss, if any, during plasmid isolation steps. The copy number of *oriII* plasmids (Figure 7) was determined similarly except that cells instead of growing in liquid cultures were obtained by washing out colonies from transformation plates directly, to avoid mutant accumulation, as described [26]. The origin fragments were first cloned in a plasmid vector driven by the *γ*-origin of plasmid R6K, and the clones were maintained in cells that supplied the cognate initiator (π) protein. The clones were electroporated into *E. coli* (BR8706) carrying pTVC499 that supplied RctB (but no π protein) and pTVC501 that supplied *parB2*.

### EMSA

The DNA probes were made from plasmids by PCR using oligonucleotides TVC286 (5′-TCCGATTACGGCACCAAATCGA-3′) and TVC287 (5′- AACGTGGATAAACTTCCTGTAAT-3′), which allowed amplification of extra 100 bp of vector sequences from each flank of the region of interest. The PCR products were labeled using 30 units T4 Polynucleotide Kinase (NEB) and 50 µCi of [γ^32^-ATP] (Perkin-Elmer) and purified by passing through G-50 columns (Roche diagnostics). Binding was done in the presence of 300 ng poly dI-dC. Other details are as described [25], except that the binding reactions were run in 0.5×TBE, which improved ParB2 binding. In Figure 5, the probe was non-radioactive and was visualized with SYBR Gold nucleic acid gel stain (Molecular Probes) at 0.5 mg/ml for 30 min. As non-specific competitor, supercoiled pUC19 DNA was used instead of poly dI-dC, as the former stayed at the top of gels and did not interfere with visualization of probe bands. The images were recorded using Fuji LAS-3000 imaging system.

### ChIP-chip assay

ChIP assay was performed as described [50]. Briefly, cells of *V. cholerae* CVC209 were cultivated in L broth at 37°C to exponential phase and cross-linked with 1% formaldehyde. After cell lysis and sonication, RctB-DNA or ParB2-DNA complexes were immunoprecipitated using RctB or ParB2 antibody, respectively. The precipitated DNA was amplified, labeled and hybridized to a custom Agilent 8 X 60K *V. cholerae* oligonucleotide microarray representing the whole genome according to the manufacturer's protocol and as described [51]. The custom tiling array contained 60 bp probes specific for both the Crick and Watson strands. The consecutive probes were separated by 140 bp in each strand and by 10 bp between the Watson and Crick strands. Data was extracted using an Agilent scanner and Agilent Feature Extraction program. Individual ChIP (Cy5) and input (Cy3) signals were first normalized with respect to total Cy5 and Cy3 signals, respectively. Fold change was calculated by dividing the normalized Cy5 signals with normalized Cy3 signals. The values are mean from three independent experiments.

### Roadblock assay

The effect of roadblock to ParB2 spreading was tested by cloning an array of five consensus P1 plasmid iterons in front of chrII origin of pTVC20, resulting in pBJH218 (Figure 8). Consensus iterons were used to avoid the P*repA* promoter present within the array of natural iterons of P1*ori*. To clone the iterons, pTVC20 was modified by creating a *Nde*I restriction enzyme site between *parS2-B* and the 39-mer within *rctA*, using QuikChange II XL site-directed mutagenesis kit (Agilent Technologies) and oligonucleotides BJH472 and BJH473. To the resulting plasmid (pBJH217), the iteron array, amplified from pALA753 [52] using oligonucleotides BJH475 and BJH476, was cloned at the *Nde*I site, resulting in pBJH218. The plasmid pair, pTVC20 and pBJH218, was used in the same genetic background as used in Figure 7, and additionally, in an otherwise isogenic host that supplied P1RepA protein from a constitutive promoter (*bla-p2* of pBR322). The P1*repA* gene and the adjoining *bla-p2* promoter was cloned in a λD69 vector and the resulting phage (λDKC311 [53]) was used to lysogenize BR8706.

The copy numbers of pTVC20 and pBJH218 were nearly identical, about four-fold lower than pTVC22 used in Figure 7, as was found earlier for pTVC20 [29]. The copy number was estimated to be about one per cell when there should be four *oriC*. The low copy number of pTVC20 and pBJH218, and the lack of an active partitioning system, made the plasmids unstable (130/130 cells lost the plasmids after seven generations of growth without selection). The effect of ParB2 was therefore checked only under selection. Young colonies (≤1 mm) from selection plates were used to inoculate LB medium with appropriate drugs (ampicillin at 50 µg/ml, chloramphenicol at 25 µg/ml and spectinomycin at 40 µg/ml) and inducers (arabinose at 0.02% and, when desired, IPTG at 100 µM), and the cultures were grown to early log phase (OD_600_∼0.1) and stored in 0.01 M MgSO_4_ in ice. For calculating generation times, the cultures were diluted to OD_600_ = 0.002 and grown to early log phase. Generation times were calculated from OD_600_ values in the range 0.02–0.2. Saturation of growth was avoided to prevent accumulation of faster growing revertants. Relative colony sizes were also determined from the MgSO_4_ suspensions on plates with and without IPTG but otherwise identical in volume and contents.

## Supporting Information

Figure S1 Spreading of ParB2 on *parS2-B*carrying plasmids in *E. coli*. A) Silencing of plasmid replication when the *parS2-B* site was present in *cis* but not when the central 39-mer was present at the same position. The sites were cloned into pGB2 to make pBJH107 and pBJH162, respectively. These plasmids and the empty vector (pGB2) were used to transform *E. coli* XL1-Blue strain containing a source of GFP-ParB2 (pBJH108), where the expression of the fusion protein is under IPTG control. Transformants were grown at 37°C for one day on LB agar plates with and without 0.5 mM of IPTG under drug selection for the presence of pGB2 plasmids. Note that under the inducing condition, cell growth was prevented only when the transformation was attempted with pGB2-*parS2-B*. B) ParB2 does not spread significantly beyond the central 39-mer. ParB2 spreading was inferred from focus formation of a GFP-ParB2 fusion protein by fluorescence microscopy. The assumption was that the appearance of fluorescent spots would indicate a localized high density of fluorescent molecules that spreading could create [28], [54]. The fragments containing *parS2-B* and the central 39-mer were cloned into pACYC184 to make TVC521 and TVC520, respectively. These plasmids together with the empty vector (pTVC158) were used to transform *E. coli* XL1-Blue strain harboring a plasmid expressing GFP-ParB2 under IPTG control (pBJH108). Cells were cultivated in L broth at 37°C to exponential phase and observed under a fluorescence microscope. Spots could be seen in the presence of *parS2-B*, as was also the finding in an earlier study [28]. In contrast, spots were not seen when the cells had the 39-mer, indicating either the GFP-ParB2 protein failed to bind to the 39-mer or, more likely, failed to elicit spreading upon binding.
(TIF)

Figure S2 Quantification of ParB2 by Western blot analysis. *E. coli* (*Ec*) extracts were from BR8706 that carried either pTVC510 containing P*lac* (lane 1), or pTVC501 containing P*lac-parB2* (lane 2,3), or pACYC184 (lane 4) or pTVC236 containing Pconst-*parB2* (lane 5). Pconst is a Δ*lacO*1 mutant of the P*trc* promoter (hence IPTG insensitive) that was cloned in pACYC184. The *V. cholerae* (*Vc*) extract was from N16961 and used as a reference for the physiological level of ParB2 made from its native promoter (Pnat). The relative [ParB2] was calculated first by accounting for the different OD equivalents of cell extracts loaded and then normalizing with respect to the level found in lane 4, defined here as 1. The cultures used in lanes 1–3 are representative of experiments in Figures 3, 7 and S3, while lanes 4–5 are representative of the experiment in Figure 2.
(TIF)

Figure S3 Silencing increases with increasing ParB2 concentration and decreases when RctB is additionally present. The origin map and the fragments (1A, 1B) used to assay silencing of P*rctA* and P*rctB* by *lacZ* reporter fusion are same as in Figure 2. The *β*-galactosidase activities were measured and plotted also as before. RctB was supplied from pTVC11 (Pbad-*rctB*) with 0 (− ; lane 4) or 0.2% Arabinose (+; lanes 5, 6). [The − sign indicates that the uninduced level of RctB was undetectable.] The RctB plasmid was absent in lanes 1–3. ParB2 was supplied at low levels from P*lac-parB2* (pTVC501) with 0 (lane 2) and 100 µM IPTG (lane 3), and at higher levels from Pconst-*parB2* (pTVC236) (lanes 4, 6). ParB2 was absent in lanes 1 and 5, where the empty vectors pTVC510 and pACYC184, respectively, were used. The activities without and with ParB2 are shown as white and grey bars, respectively. Protein levels shown are relative to the wild type level present in N16961 (Fig. S2 of [26] for RctB and Fig. S2 for ParB2). Note that at the lower level ParB2 down-regulates P*rctA* but not P*rctB* and, at the higher level down regulates both the promoters, indicating that the span of silencing increases with ParB2 concentration. The strength of silencing also increases at higher ParB2 concentrations (P*rctA* in lanes 2–4). RctB can overcome partially the silencing effect on both the promoters (lanes 4 vs. 6: the lanes are also shown in insets where the ordinate scale is expanded). The copy number of plasmids with origin fragments was around 60 per cell assuming there are four *oriC* copies in newborn *E. coli* cells in LB.
(TIF)

Figure S4 ParB2, unlike RctB, does not require the central 39-mer to be intact for specific binding. The 39-mer has three elements, composed of two direct repeats [bold letters and called A (left) and B (right) boxes] that flank a dyad symmetric AT-rich spacer (arrows in DNA #1). The elements were tested individually and in combination for their ability to bind ParB2 by EMSA (DNA #2–6). Unlike RctB, whose binding to 39-mer requires all three elements [lower panel; [26]], ParB2 could bind without one of the direct repeats (DNA #5 and 6). The AT-rich spacer, which shows some homology to the consensus *parS2* site (the top DNA sequence), was necessary but not its dyad symmetric feature (DNA #1 and #12). The DNA samples #1–12 were obtained from plasmids pTVC222, −132, −190, −120, −119, −156, −182, −184, −181, 330, −332 and −525. All had 100 bp vector flanks, whereas the negative control consisted of the flanks only (from pTVC243 where the 39-mer sequences were cloned). DNA fragments [2 nM each] were subjected to EMSA with purified RctB and ParB2, each at two concentrations: 3 nM (+) and 30 nM (++) for RctB, and 0.7 µM (+) and 1.4 µM (++) for ParB2.
(TIF)

Figure S5 DNase I footprinting of the central 39-mer by ParB2 and RctB. The DNA-protein complexes after treatment with DNase I were purified using EMSA gels and analysed in 6% sequencing gels. Both upper and lower strands of DNA were analyzed. Samples in lanes marked GATC were made by the Sanger method, using the same primers that were used to make DNA fragments used for footprinting. The bands in these lanes serve as length standards. Lane 1 had no ParB2 or RctB, lane 2 had 1 µM ParB2 and lane 3 had 0.3 µM RctB. The GC-rich direct repeats of the 39-mer (bold letter sequences named A and B boxes below the autoradiogram) are present in brackets, marked A and B, which border the AT-rich inverted repeat (inverted arrows). The red star indicates the base(s) protected by ParB2 which are near the border between the A box and the inverted repeats. Short horizontal arrows mark DNase I hypersensitive sites, which are located at the center of the inverted repeats. Note that in contrast to ParB2, which affected a couple of positions only, the entire 39-mer was protected by RctB (long brackets alongside lane 3). In fact, the protection extended beyond the B box by 12 nucleotides on the top strand and eight nucleotides in the bottom strand. The bottom panel shows the sequences of the 39-mer and its natural flanks present in the fragment used in footprinting.
(TIF)

Figure S6 The central 39-mer is not a centromere. The centromeric function of the 39-mer was tested by cloning it into an unstable miniF plasmid (pDAG203) and supplying ParA2 and ParB2 proteins in *trans* from a compatible plasmid (pTVC508), where the *parAB2* genes were under IPTG control. Introduction of the 39-mer, as opposed to the *parS2-B* (positive control) to pDAG203, however, did not improve the stability (red vs. blue lines). Note that under our growth conditions, the empty vector, pDAG203 (black line), was reasonably stable to start with, as was found earlier [55]. Also note that the addition of 100 µM IPTG, slightly destabilized the positive control plasmid, most likely because of silencing, but stabilized the 39-mer carrying plasmid. However, the empty vector was also stabilized equally. The reason for this IPTG effect has not been studied. The data points are from three repeat experiments of two independent cultures in each of the three strains tested. Because all the miniF plasmids were present in all cells (in 100/100 cells tested in all three cases) at the start of the experiment, as they were grown previously under selection, data points were fit to exponential curves with an intercept at zero generation and 100% plasmid retention. These plasmids were equally unstable in cells without the Par proteins.
(TIF)

Figure S7 Deletion of *parS2-B* does not prevent ParB2 spreading into the chrII origin in *V. cholerae*. The *parS2-B* site of N16961 (CVC2335) was exchanged for a FRT site to generate an otherwise isogenic strain (CVC2336). ChIP assay was performed as described using antibody against ParB2 [50]. The amount of precipitated DNA compared to the total DNA was determined by qPCR. The precipitated DNA in the regions of *parB2*, *parA2*, *parS2-B*, 39-mer, 12- and 11-mer, *rctB* promoter, *rctB* gene, and *parS1* (one of the *parS* sites belonging to chrI and used here as a negative control) was determined from three independent experiments. Overall binding of ParB2 in the origin region was reduced upon deletion of the *parS2-B* site but it was still significant, and most likely originated from one of the neighboring *parS2* sites about 6 kb away (Fig. 1, bottom panel). Preferential binding to the 39-mer was not conspicuous in these studies, suggesting that the region is occupied mostly by spreading in the cell cycle of *V. cholerae*.
(TIF)

Figure S8 Interactions between ParB2 and RctB by the bacterial two-hybrid assay and co-immunoprecipitation. The two-hybrid assay was performed using the bacterial adenylate cyclase two-hybrid (BACTH) system (EUK001, Euromedex, France) as described [22]. The *parB2* and *rctB* genes of *V. cholerae* N16961 were cloned into pKT25 and pUT18C to make bait and prey plasmids, respectively (Table S1). Pairs of bait and prey plasmids were used to transform *E. coli* BTH101 (CVC1837) cells, and the transformants were cultivated at 30°C for 1 or 2 days on LB agar plate containing 0.5 mM IPTG and 40 µg/ml X-gal. For co-immunoprecipitation, the cells were *V. cholerae* wild type (CVC209) or *E. coli* BR8706 harboring pTVC499 and pTVC501. ParB2 and RctB were induced from these plasmids using100 µM IPTG and 0.2% arabinose, respectively. The cells were lysed by sonication, and from the lysate, proteins of interest were immunoprecipitated using anti-ParB2 and anti-RctB polyclonal antibodies and Dynabeads protein G (100.03D, Invitrogen) according to the manufacturer's protocol. The precipitated proteins were detected by Western blotting as described for Fig. S2. No significant ParB2-RctB interactions could be detected in either of the assays. An earlier study used the bacterial two-hybrid assay to draw the same conclusion [23].
(TIF)

Figure S9 Effect of ParA2 on binding and spreading of ParB2 in *E. coli*. These were tested using promoters P*rctA*, P*rctB* and P*repA*, the latter a foreign promoter. P*repA* was fused to the central 39-mer, as in pTVC529, and the promoter was close enough that ParB2 binding to the 39-mer could reduce the promoter activity. Plasmids carrying the origin fragments 1A and 2A for testing P*rctA* activity, and fragments 1B and 2B for testing P*rctB* activity were same as in Figure 2. ParB2, either alone or together with ParA2, was supplied from P*lac* of plasmids pTVC501 or pTVC508, respectively. The white and the grey bars represent activities of P*rctA*, P*rctB* and P*repA* either with no induction or induction of P*lac* with 250 µM IPTG, respectively. The results show that ParA2 does not affect repression of P*repA* by ParB2, implying no effect on the direct binding of ParB2 to the central 39-mer. ParA2, however, increased silencing of P*rctA* present in 1A, as the repression of the promoter increased from 68% to 83%. No effect of ParA2 was evident on fragment 1B, which suggests that *parS2-B* needs to be present *in cis* and close to the promoter. Western blotting of ParB2 is shown at the bottom, which indicates that the increased silencing of P*rctA* in the presence of ParA2 is not due to increased level of ParB2 (note comparable band intensities in P*lac-parB2* and P*lac-parAB2* carrying cells).
(TIF)

Table S1 Bacterial strains and plasmids used in this study.
(DOCX)

Table S2 Primers used in this study.
(DOCX)

Text S1 Supporting Materials and Methods.
(DOC)
