# Supplementary figures and images for: Evidence for Two Different Regulatory Mechanisms Linking Replication and Segregation of Vibrio cholerae Chromosome II
Source: PLoS Genet. 2013 Jun 20;9(6):e1003579. doi: 10.1371/journal.pgen.1003579 (PMC3688505; doi:10.1371/journal.pgen.1003579)

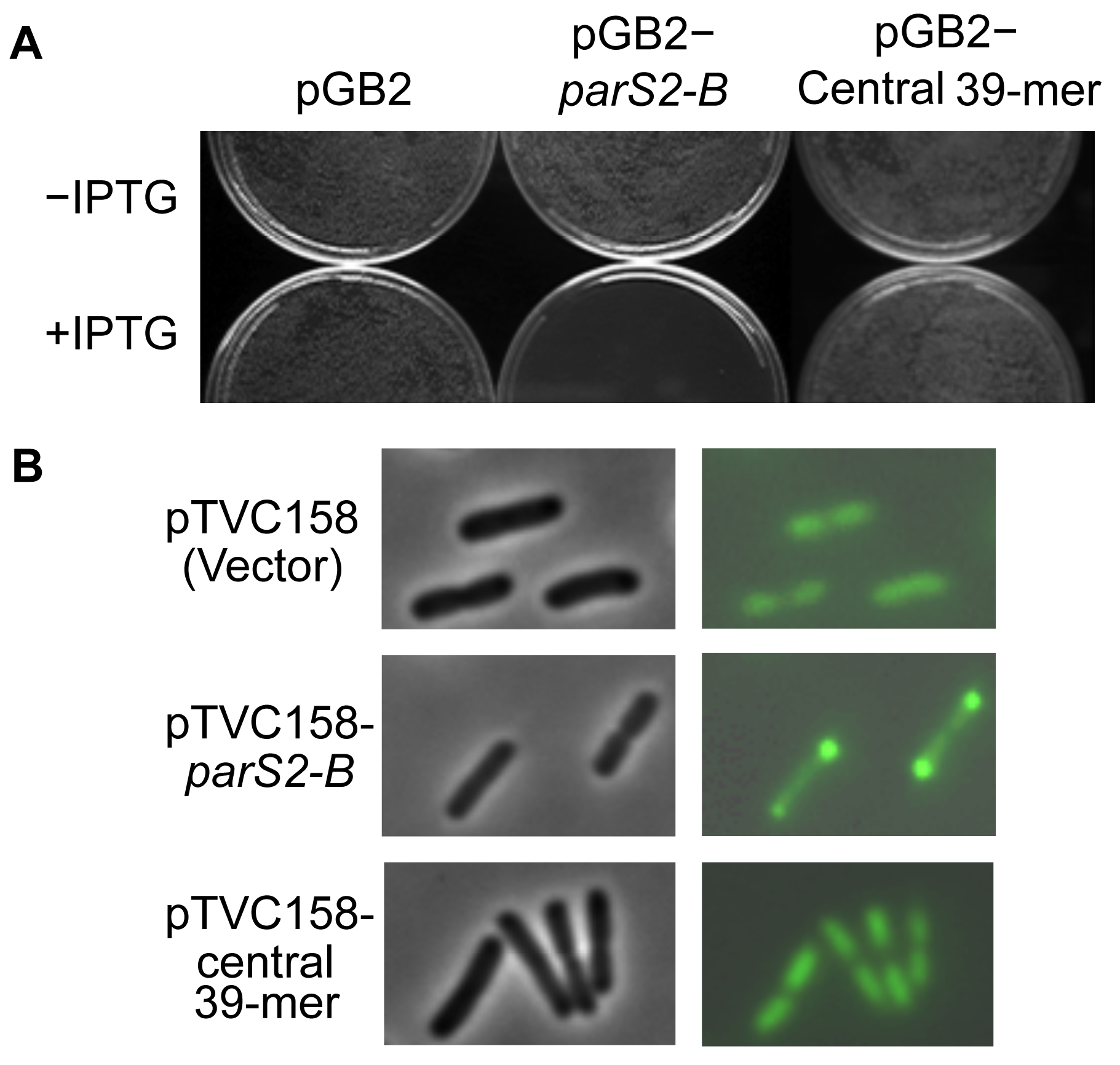

Supplement: Figure S1 — Spreading of ParB2 on parS2-Bcarrying plasmids in E. coli. A) Silencing of plasmid replication when the parS2-B site was present in cis but not when the central 39-mer was present at the same position. The sites were cloned into pGB2 to make pBJH107 and pBJH162, respectively. These plasmids and the empty vector (pGB2) were used to transform E. coli XL1-Blue strain containing a source of GFP-ParB2 (pBJH108), where the expression of the fusion protein is under IPTG control. Transformants were grown at 37°C for one day on LB agar plates with and without 0.5 mM of IPTG under drug selection for the presence of pGB2 plasmids. Note that under the inducing condition, cell growth was prevented only when the transformation was attempted with pGB2-parS2-B. B) ParB2 does not spread significantly beyond the central 39-mer. ParB2 spreading was inferred from focus formation of a GFP-ParB2 fusion protein by fluorescence microscopy. The assumption was that the appearance of fluorescent spots would indicate a localized high density of fluorescent molecules that spreading could create [28], [54]. The fragments containing parS2-B and the central 39-mer were cloned into pACYC184 to make TVC521 and TVC520, respectively. These plasmids together with the empty vector (pTVC158) were used to transform E. coli XL1-Blue strain harboring a plasmid expressing GFP-ParB2 under IPTG control (pBJH108). Cells were cultivated in L broth at 37°C to exponential phase and observed under a fluorescence microscope. Spots could be seen in the presence of parS2-B, as was also the finding in an earlier study [28]. In contrast, spots were not seen when the cells had the 39-mer, indicating either the GFP-ParB2 protein failed to bind to the 39-mer or, more likely, failed to elicit spreading upon binding. (TIF) [file pgen.1003579.s001.tif]

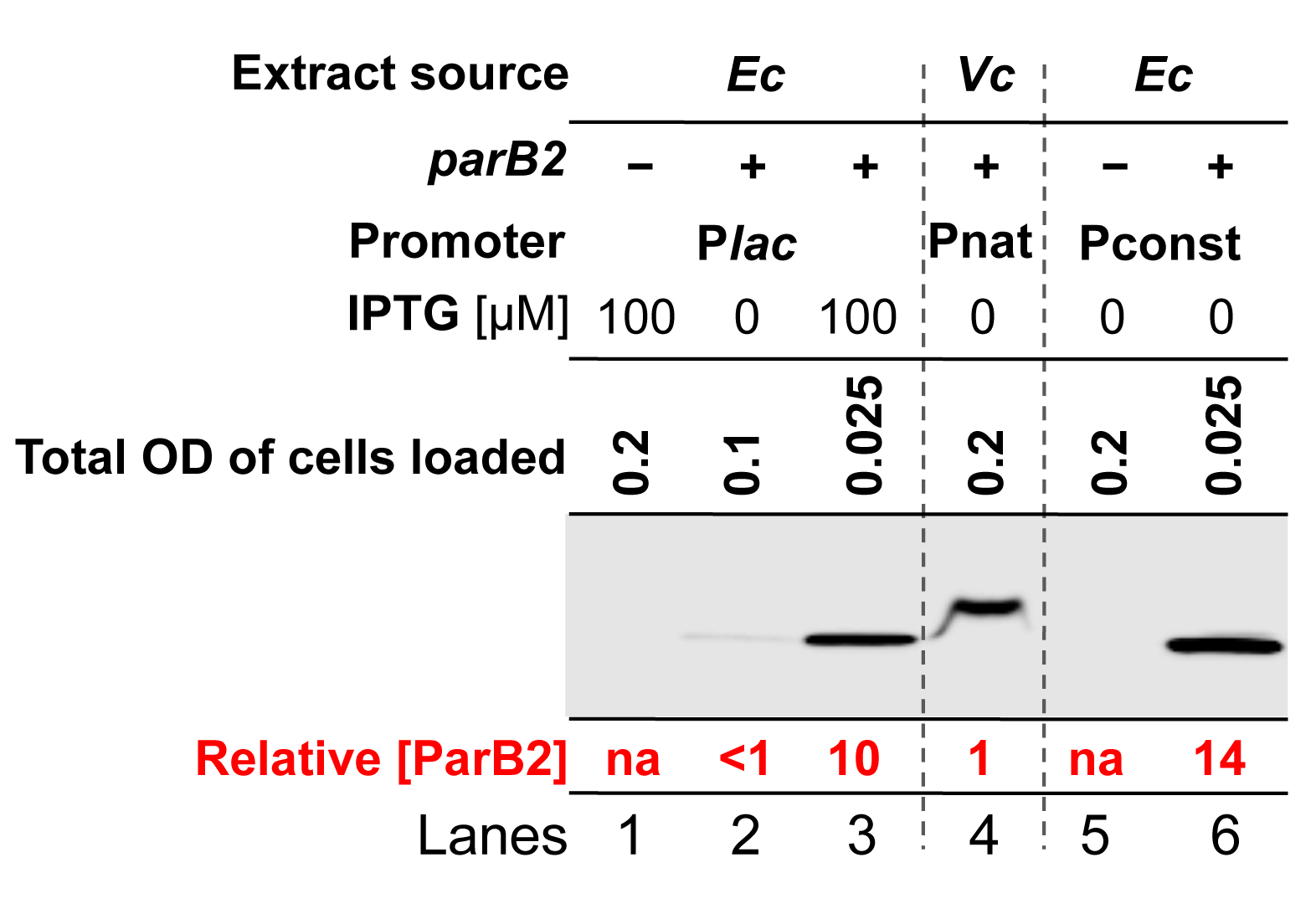

Supplement: Figure S2 — Quantification of ParB2 by Western blot analysis. E. coli (Ec) extracts were from BR8706 that carried either pTVC510 containing Plac (lane 1), or pTVC501 containing Plac-parB2 (lane 2,3), or pACYC184 (lane 4) or pTVC236 containing Pconst-parB2 (lane 5). Pconst is a ΔlacO1 mutant of the Ptrc promoter (hence IPTG insensitive) that was cloned in pACYC184. The V. cholerae (Vc) extract was from N16961 and used as a reference for the physiological level of ParB2 made from its native promoter (Pnat). The relative [ParB2] was calculated first by accounting for the different OD equivalents of cell extracts loaded and then normalizing with respect to the level found in lane 4, defined here as 1. The cultures used in lanes 1–3 are representative of experiments in Figures 3, 7 and S3, while lanes 4–5 are representative of the experiment in Figure 2. (TIF) [file pgen.1003579.s002.tif]

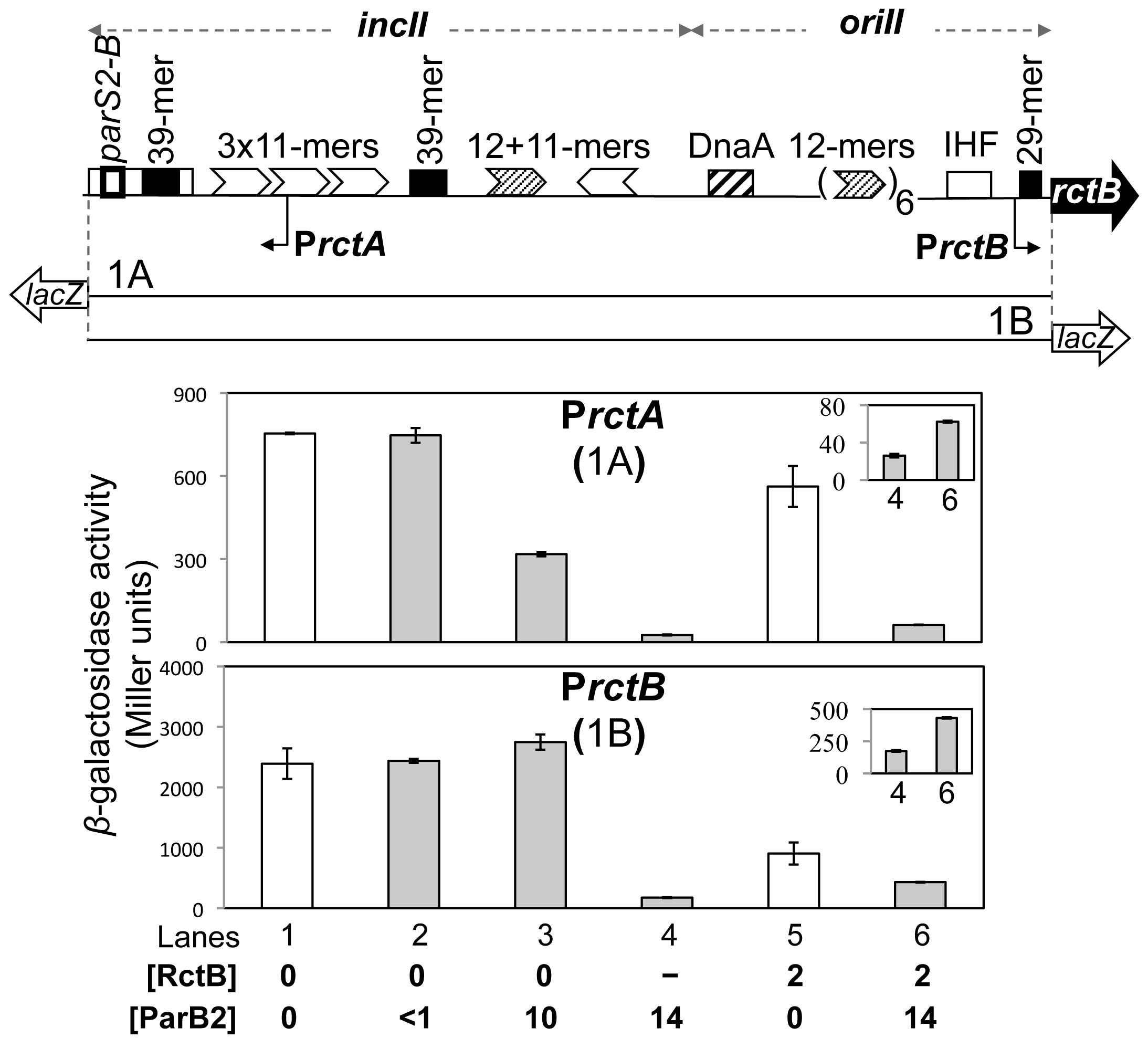

Supplement: Figure S3 — Silencing increases with increasing ParB2 concentration and decreases when RctB is additionally present. The origin map and the fragments (1A, 1B) used to assay silencing of PrctA and PrctB by lacZ reporter fusion are same as in Figure 2. The β-galactosidase activities were measured and plotted also as before. RctB was supplied from pTVC11 (Pbad-rctB) with 0 (− ; lane 4) or 0.2% Arabinose (+; lanes 5, 6). [The − sign indicates that the uninduced level of RctB was undetectable.] The RctB plasmid was absent in lanes 1–3. ParB2 was supplied at low levels from Plac-parB2 (pTVC501) with 0 (lane 2) and 100 µM IPTG (lane 3), and at higher levels from Pconst-parB2 (pTVC236) (lanes 4, 6). ParB2 was absent in lanes 1 and 5, where the empty vectors pTVC510 and pACYC184, respectively, were used. The activities without and with ParB2 are shown as white and grey bars, respectively. Protein levels shown are relative to the wild type level present in N16961 (Fig. S2 of [26] for RctB and Fig. S2 for ParB2). Note that at the lower level ParB2 down-regulates PrctA but not PrctB and, at the higher level down regulates both the promoters, indicating that the span of silencing increases with ParB2 concentration. The strength of silencing also increases at higher ParB2 concentrations (PrctA in lanes 2–4). RctB can overcome partially the silencing effect on both the promoters (lanes 4 vs. 6: the lanes are also shown in insets where the ordinate scale is expanded). The copy number of plasmids with origin fragments was around 60 per cell assuming there are four oriC copies in newborn E. coli cells in LB. (TIF) [file pgen.1003579.s003.tif]

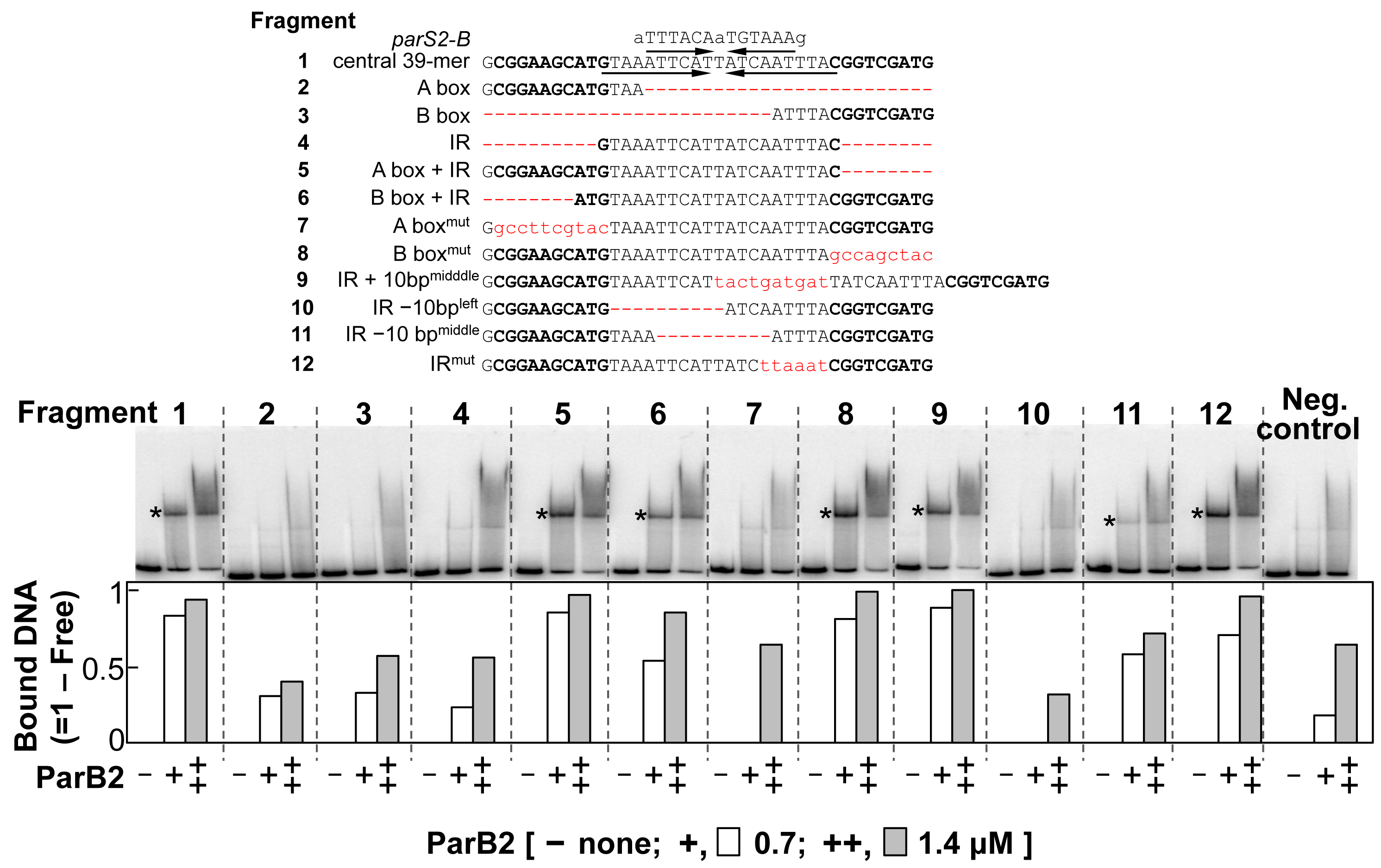

Supplement: Figure S4 — ParB2, unlike RctB, does not require the central 39-mer to be intact for specific binding. The 39-mer has three elements, composed of two direct repeats [bold letters and called A (left) and B (right) boxes] that flank a dyad symmetric AT-rich spacer (arrows in DNA #1). The elements were tested individually and in combination for their ability to bind ParB2 by EMSA (DNA #2–6). Unlike RctB, whose binding to 39-mer requires all three elements [lower panel; [26]], ParB2 could bind without one of the direct repeats (DNA #5 and 6). The AT-rich spacer, which shows some homology to the consensus parS2 site (the top DNA sequence), was necessary but not its dyad symmetric feature (DNA #1 and #12). The DNA samples #1–12 were obtained from plasmids pTVC222, −132, −190, −120, −119, −156, −182, −184, −181, 330, −332 and −525. All had 100 bp vector flanks, whereas the negative control consisted of the flanks only (from pTVC243 where the 39-mer sequences were cloned). DNA fragments [2 nM each] were subjected to EMSA with purified RctB and ParB2, each at two concentrations: 3 nM (+) and 30 nM (++) for RctB, and 0.7 µM (+) and 1.4 µM (++) for ParB2. (TIF) [file pgen.1003579.s004.tif]

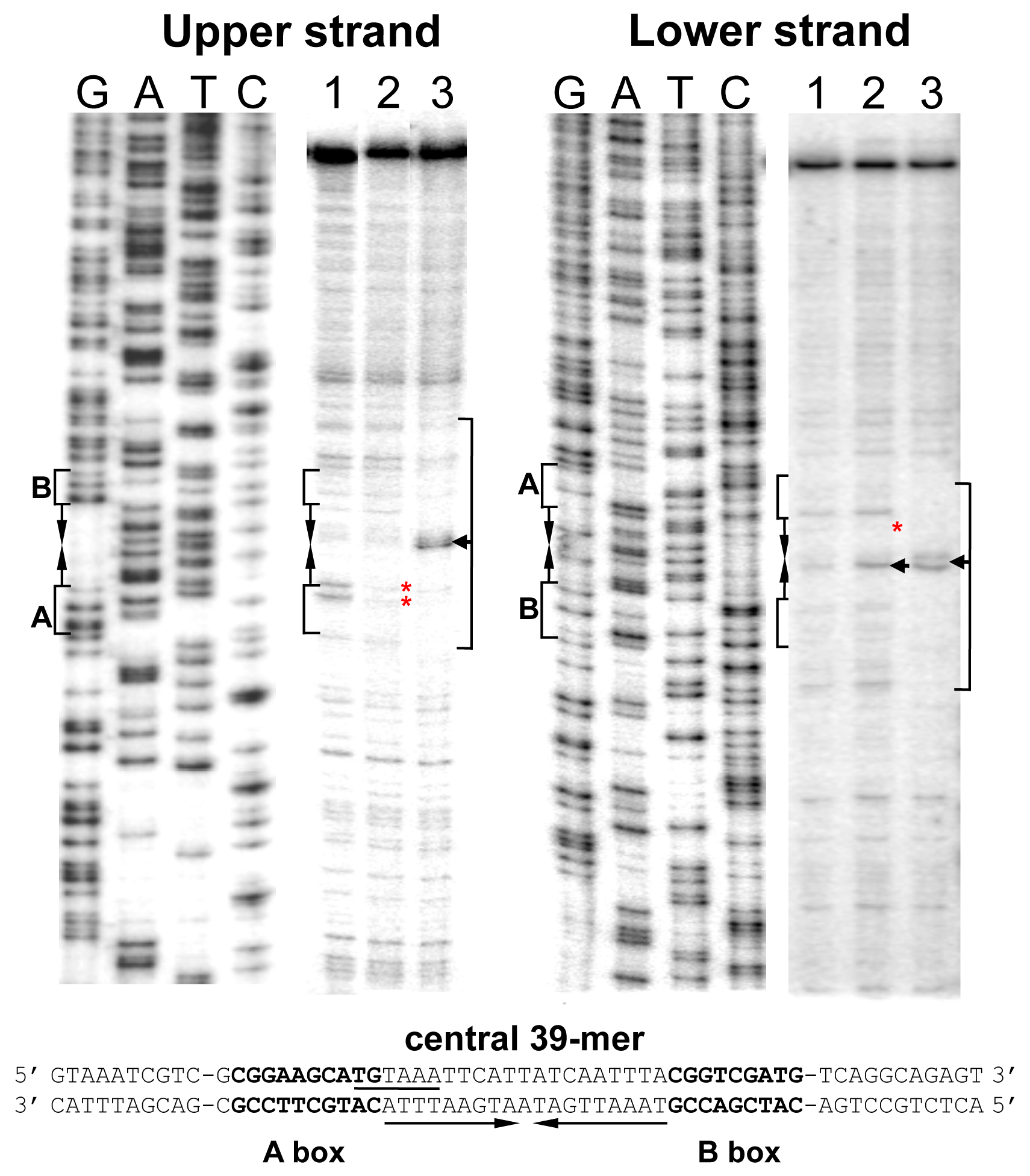

Supplement: Figure S5 — DNase I footprinting of the central 39-mer by ParB2 and RctB. The DNA-protein complexes after treatment with DNase I were purified using EMSA gels and analysed in 6% sequencing gels. Both upper and lower strands of DNA were analyzed. Samples in lanes marked GATC were made by the Sanger method, using the same primers that were used to make DNA fragments used for footprinting. The bands in these lanes serve as length standards. Lane 1 had no ParB2 or RctB, lane 2 had 1 µM ParB2 and lane 3 had 0.3 µM RctB. The GC-rich direct repeats of the 39-mer (bold letter sequences named A and B boxes below the autoradiogram) are present in brackets, marked A and B, which border the AT-rich inverted repeat (inverted arrows). The red star indicates the base(s) protected by ParB2 which are near the border between the A box and the inverted repeats. Short horizontal arrows mark DNase I hypersensitive sites, which are located at the center of the inverted repeats. Note that in contrast to ParB2, which affected a couple of positions only, the entire 39-mer was protected by RctB (long brackets alongside lane 3). In fact, the protection extended beyond the B box by 12 nucleotides on the top strand and eight nucleotides in the bottom strand. The bottom panel shows the sequences of the 39-mer and its natural flanks present in the fragment used in footprinting. (TIF) [file pgen.1003579.s005.tif]

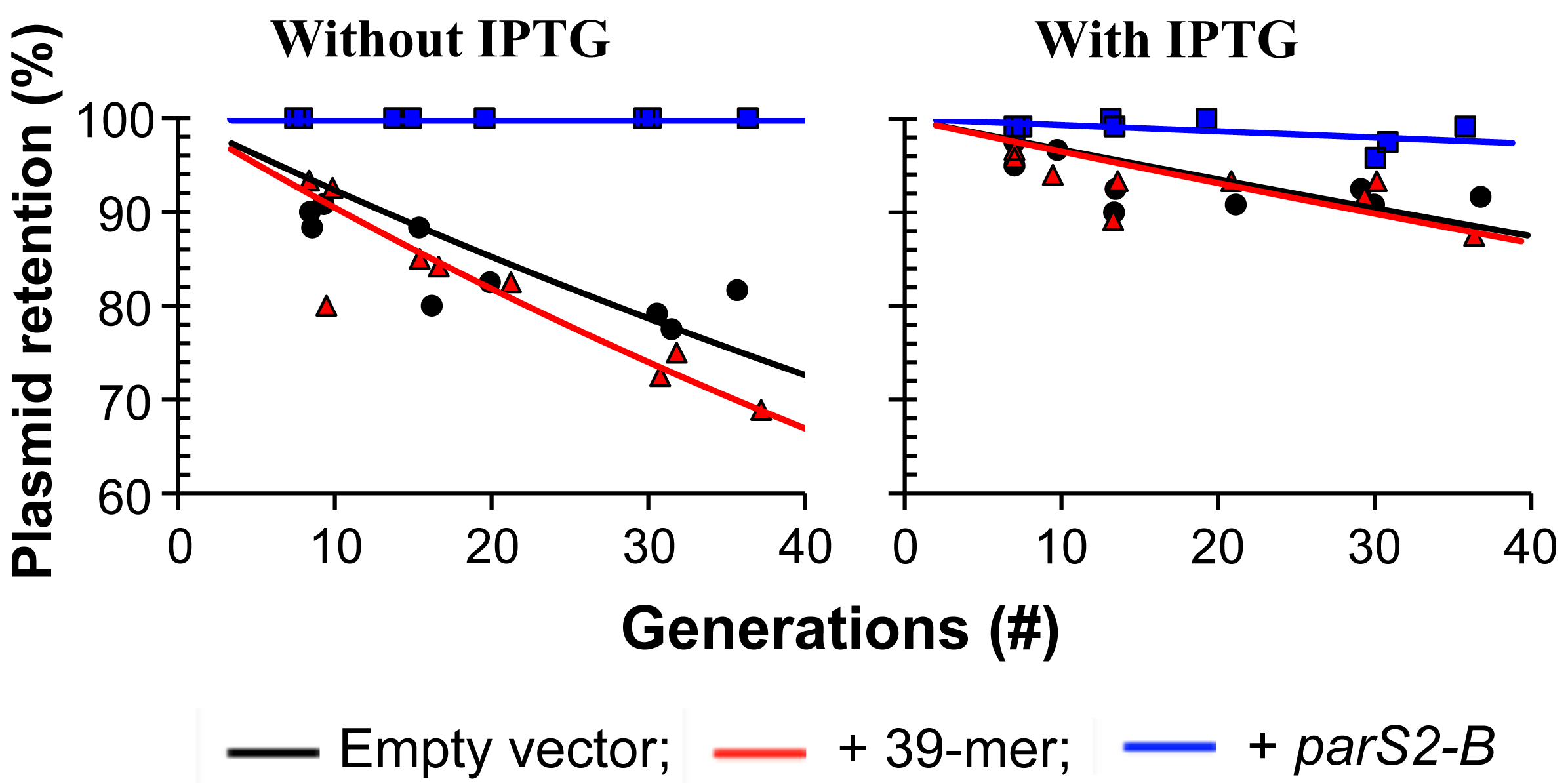

Supplement: Figure S6 — The central 39-mer is not a centromere. The centromeric function of the 39-mer was tested by cloning it into an unstable miniF plasmid (pDAG203) and supplying ParA2 and ParB2 proteins in trans from a compatible plasmid (pTVC508), where the parAB2 genes were under IPTG control. Introduction of the 39-mer, as opposed to the parS2-B (positive control) to pDAG203, however, did not improve the stability (red vs. blue lines). Note that under our growth conditions, the empty vector, pDAG203 (black line), was reasonably stable to start with, as was found earlier [55]. Also note that the addition of 100 µM IPTG, slightly destabilized the positive control plasmid, most likely because of silencing, but stabilized the 39-mer carrying plasmid. However, the empty vector was also stabilized equally. The reason for this IPTG effect has not been studied. The data points are from three repeat experiments of two independent cultures in each of the three strains tested. Because all the miniF plasmids were present in all cells (in 100/100 cells tested in all three cases) at the start of the experiment, as they were grown previously under selection, data points were fit to exponential curves with an intercept at zero generation and 100% plasmid retention. These plasmids were equally unstable in cells without the Par proteins. (TIF) [file pgen.1003579.s006.tif]

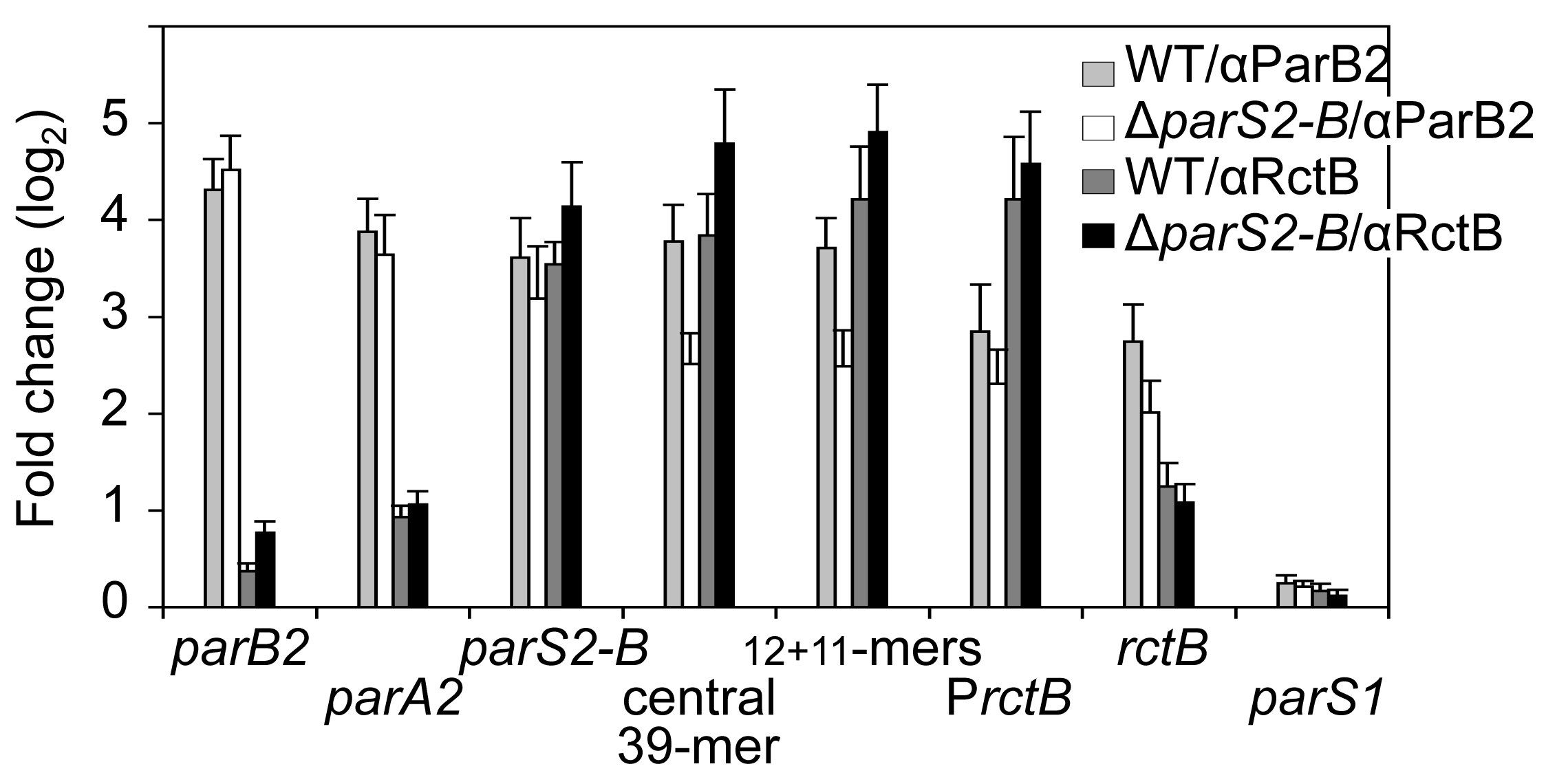

Supplement: Figure S7 — Deletion of parS2-B does not prevent ParB2 spreading into the chrII origin in V. cholerae. The parS2-B site of N16961 (CVC2335) was exchanged for a FRT site to generate an otherwise isogenic strain (CVC2336). ChIP assay was performed as described using antibody against ParB2 [50]. The amount of precipitated DNA compared to the total DNA was determined by qPCR. The precipitated DNA in the regions of parB2, parA2, parS2-B, 39-mer, 12- and 11-mer, rctB promoter, rctB gene, and parS1 (one of the parS sites belonging to chrI and used here as a negative control) was determined from three independent experiments. Overall binding of ParB2 in the origin region was reduced upon deletion of the parS2-B site but it was still significant, and most likely originated from one of the neighboring parS2 sites about 6 kb away (Fig. 1, bottom panel). Preferential binding to the 39-mer was not conspicuous in these studies, suggesting that the region is occupied mostly by spreading in the cell cycle of V. cholerae. (TIF) [file pgen.1003579.s007.tif]

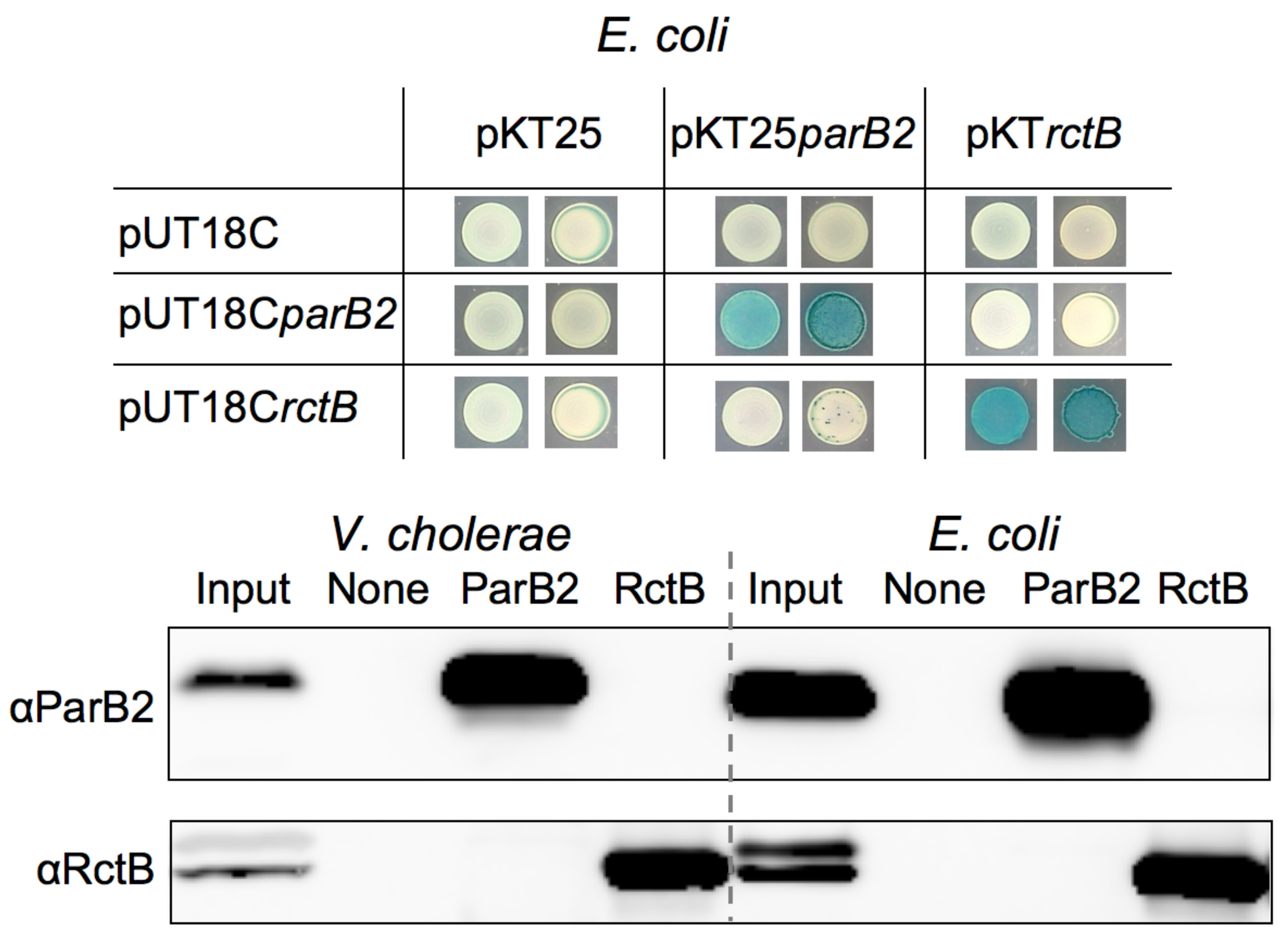

Supplement: Figure S8 — Interactions between ParB2 and RctB by the bacterial two-hybrid assay and co-immunoprecipitation. The two-hybrid assay was performed using the bacterial adenylate cyclase two-hybrid (BACTH) system (EUK001, Euromedex, France) as described [22]. The parB2 and rctB genes of V. cholerae N16961 were cloned into pKT25 and pUT18C to make bait and prey plasmids, respectively (Table S1). Pairs of bait and prey plasmids were used to transform E. coli BTH101 (CVC1837) cells, and the transformants were cultivated at 30°C for 1 or 2 days on LB agar plate containing 0.5 mM IPTG and 40 µg/ml X-gal. For co-immunoprecipitation, the cells were V. cholerae wild type (CVC209) or E. coli BR8706 harboring pTVC499 and pTVC501. ParB2 and RctB were induced from these plasmids using100 µM IPTG and 0.2% arabinose, respectively. The cells were lysed by sonication, and from the lysate, proteins of interest were immunoprecipitated using anti-ParB2 and anti-RctB polyclonal antibodies and Dynabeads protein G (100.03D, Invitrogen) according to the manufacturer's protocol. The precipitated proteins were detected by Western blotting as described for Fig. S2. No significant ParB2-RctB interactions could be detected in either of the assays. An earlier study used the bacterial two-hybrid assay to draw the same conclusion [23]. (TIF) [file pgen.1003579.s008.tif]

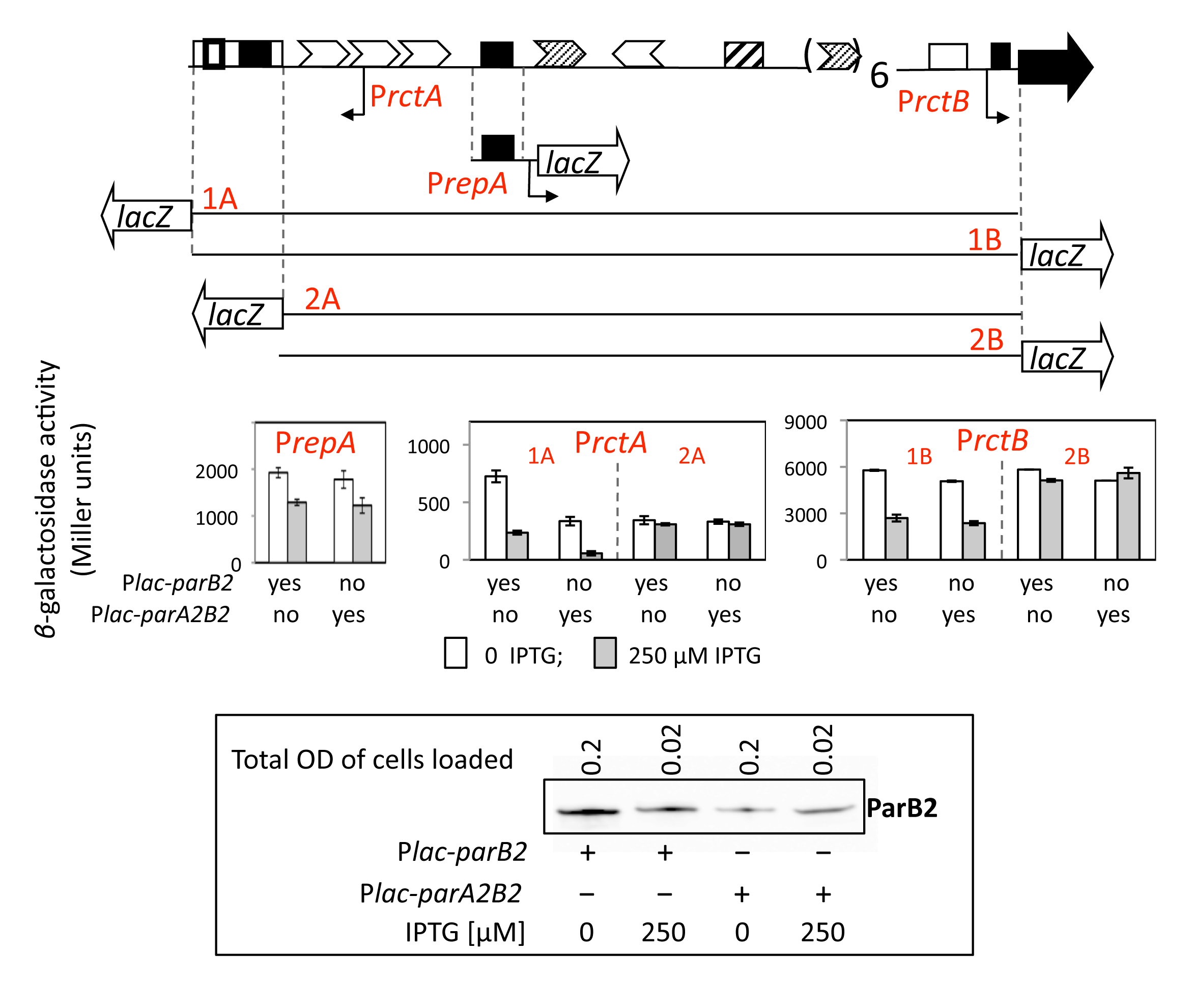

Supplement: Figure S9 — Effect of ParA2 on binding and spreading of ParB2 in E. coli. These were tested using promoters PrctA, PrctB and PrepA, the latter a foreign promoter. PrepA was fused to the central 39-mer, as in pTVC529, and the promoter was close enough that ParB2 binding to the 39-mer could reduce the promoter activity. Plasmids carrying the origin fragments 1A and 2A for testing PrctA activity, and fragments 1B and 2B for testing PrctB activity were same as in Figure 2. ParB2, either alone or together with ParA2, was supplied from Plac of plasmids pTVC501 or pTVC508, respectively. The white and the grey bars represent activities of PrctA, PrctB and PrepA either with no induction or induction of Plac with 250 µM IPTG, respectively. The results show that ParA2 does not affect repression of PrepA by ParB2, implying no effect on the direct binding of ParB2 to the central 39-mer. ParA2, however, increased silencing of PrctA present in 1A, as the repression of the promoter increased from 68% to 83%. No effect of ParA2 was evident on fragment 1B, which suggests that parS2-B needs to be present in cis and close to the promoter. Western blotting of ParB2 is shown at the bottom, which indicates that the increased silencing of PrctA in the presence of ParA2 is not due to increased level of ParB2 (note comparable band intensities in Plac-parB2 and Plac-parAB2 carrying cells). (TIF) [file pgen.1003579.s009.tif]
