# Supplementary material for: Evidence for Two Different Regulatory Mechanisms Linking Replication and Segregation of Vibrio cholerae Chromosome II
Source: PLoS Genet. 2013 Jun 20;9(6):e1003579. doi: 10.1371/journal.pgen.1003579 (PMC3688505; doi:10.1371/journal.pgen.1003579)
Supplement: Table S1 — Bacterial strains and plasmids used in this study. (DOCX) [file pgen.1003579.s010.docx]

**Table S1.** Bacterial strains and plasmids used in this study.

| **Strains** | **Relevant characteristics** | **Source / Used in** |
| --- | --- | --- |
| ***E. coli*** |  |  |
| BR2846 | K-12 *recA1* Δ(*argF-lac*)*U169* Strain for cloning in pMLB1109 | [1] |
| BR8706 | Stbl2 Δ(*araFGH*) Δ*araEp* P_CP18_-*araE*; *araE* under constitutive CP18 promoter | [2] |
| CVC553 | DH5Δ*lac*(λ*pir*), where λ*pir*=λDKC370; Strain for maintaining R6K*oriγ* plasmids | [3] |
| CVC1501 | BR8706(λ*pir*), where λ*pir*=λDKC370; Strain for measuring *β*-galactosidase activities from R6K*oriγ* driven plasmids | This study |
| CVC1837 | (=BTH101) F^-^ *cya-99* *araD139 galE15 galK16 rpsL1* (Str^R^) *hsdR2 mcrA1 mcrB1* | Euromedex |
| CVC2535 | BR8706(λDKC311); supplies P1 RepA constitutively from *bla-p2* of pBR322 | Fig. 8 |
| DH5α | Strain for cloning | Invitrogen |
| XL1-Blue | Strain for cloning | Agilent Technologies |
| ***V. cholerae*** |  |  |
| CVC209 | N16961 El Tor (*hapR*^fs^); Str^R^ | M. Waldor |
| CVC2335 | (=Blokesch#2) N16961 El Tor (*hapR*^fs^); Str^R^ | This study |
| CVC2336 | (=Blokesch#1875) CVC2335 *ΔparS2-B*::FRT (with a GAAG → GACG change in FRT) *hapR*^fs^; Str^R^, Ap^S^, Rif^S^ | This study |
|  |  |  |
| **Plasmids** |  |  |
| pACYC184 | Cloning vector, p15A*ori*; Cm^R^ | NEB; Figs. 2, S2 and S3 |
| pALA753 | pUC19 with an array of five consensus P1 iterons; Ap^R^ | [4] |
| pAS1 | P*rctB* (nt 1049-1133)* fused to *lacZ* in pMLB1109; Ap^R^ | [5,6,7]; Fig. 2 |
| pBJH92 | *parB2* (nt 1070989-1070021) in pKT25; Km^R^ | Fig. S8 |
| pBJH93 | *rctB* (nt 1118-3115) in pKT25; Km^R^ | Fig. S8 |
| pBJH94 | *parB2* (nt 1070989-1070021) in pUT18C; Ap^R^ | Fig. S8 |
| pBJH95 | *rctB* (nt 1118-3115) in pUT18C; Ap^R^ | Fig. S8 |
| pBJH107 | *parS2-B* (nt 118-132) in pGB2; Sp^R^ | Fig. S1*A* |
| pBJH108 | *parB2* (nt 1070989-1070021) fused to *gfp* of pDSW209 ; Ap^R^ | S. Austin; Fig. S1*B* |
| pBJH162 | 39-mer (nt 449-487) in pGB2; Sp^R^ | Fig. S1*A* |
| pBJH180 | *parS2-B* (nt 79-142) in pDAG203; Cm^R^ | Fig. S6 |
| pBJH181 | 39-mer (nt 440-520) in pDAG203; Cm^R^ | Fig. S6 |
| pBJH200 | pTVC243 + *parS2-B* (nt 118-132); Cm^R^ | Fig. 5 |
| pBJH201 | pTVC243 + TGTAAA; Cm^R^ | Fig. 5 |
| pBJH216 | pTVC243 + *oriII* (nt 449-462) + A; Cm^R^ | Fig. 5 |
| pBJH217 | pTVC20 + *Nde*I site between *parS2-B* and *39-mer*; Ap^R^ | Fig. 8 |
| pBJH218 | pBJH217 + five P1 iterons from pALA753; Ap^R^ | Fig. 8 |
| pBJH221 | pTVC243 + T + *oriII* (nt 459-479); Cm^R^ | Fig. 5 |
| pDAG203 | miniF plasmid; Cm^R^ | [8] |
| pGB2 | pSC101 derivative; Sp^R^ | [9]; Fig. S1*A* |
| pKT25 | Bacterial two-hybrid bait vector; Km^R^ | Euromedex; Fig. S8 |
| pMLB1109 | Source of promoter-less *lacZ* gene; Ap^R^ | M. Berman |
| pPS69 | pBR322*ori*-plasmid with a mutation in P*trc* promoter converting it to a constitutive promoter; Ap^R^ | [10] |
| pRKG211 | *parB2* (nt 1070989-1070021) in pTXB1; Ap^R^ | R. Ghosh |
| pTVC11 | *rctB* (nt 1118-3115) under P_BAD_ in pSC101*ori* plasmid; Sp^R^ | [11]; Fig. S3 |
| pTVC17 | Same as pTVC11, but *rctB* is under *lacI^q^-Plac* | [12]; Used for cloning |
| pTVC20 | *oriII* (nt 109 - 1133) in R6K *oriγ* plasmid, pGP704; Ap^R^ | [12]; Fig. 8 |
| pTVC22 | Same as pTVC20, except *oriII* (nt 253 - 1133); Ap^R^ | [12]; Fig. 7 |
| pTVC25 | Same as pTVC20, except *oriII* (nt 441 - 1133); Ap^R^ | Fig. 7 |
| pTVC26 | Same as pTVC20, except *oriII* (nt 468 - 1133); Ap^R^ | Fig. 7 |
| pTVC31 | Same as pTVC20, except *oriII* (nt 775 - 1133); Ap^R^ | [12]; Fig. 7 |
| pTVC61 | pBR322 derivative carrying MCS in a transcription-free region of phage lambda; Cm^R^ | [12]; Used for cloning |
| pTVC119 | pTVC243 + *oriII* (nt 449-479); Cm^R^ | Fig. S4 |
| pTVC120 | pTVC243 + *oriII* (nt 459-479); Cm^R^ | Fig. S4 |
| pTVC122 | *oriII* (nt 109 - 1133) in R6K *oriγ* plasmid with P*rctA* fused to a promoter-less *lacZ* gene from pMLB1109; Ap^R^ | Figs. 2, S3, S9 |
| pTVC123 | Same as pTVC122 except for *oriII* (nt 253 – 1133); Ap^R^ | Figs. 2, S9 |
| pTVC124 | P*rctA* (nt 377-316) fused to *lacZ* in pMLB1109; Ap^R^ | Fig. 2 |
| pTVC132 | pTVC243 + *oriII* (nt 449-462); Cm^R^ | Fig. S4 |
| pTVC139 | pTVC243 + *oriII* (nt 966-1065); Cm^R^ | Fig. 4 |
| pTVC156 | pTVC243 + *oriII* (nt 456-487); Cm^R^ | Fig. S4 |
| pTVC158 | Transcription-free region from pRLM167 in pACYC184; Cm^R^ | [3]; Fig. S6 |
| pTVC181 | pTVC61 + 39-mer (nt 449-487) with 10 bp-insertion (5’-ATTATTATAA-3’) at position nt 468; Cm^R^ | [3]; Fig. S4 |
| pTVC182 | pTVC61 + 39-mer (nt 449-487) with A-box (5’-CGGAAGCATG-3’) changed to 5’-GCCTTCGTAC-3’ (GC⇒CG; AT⇒TA); Cm^R^ | [3]; Fig. S4 |
| pTVC184 | pTVC61 + 39-mer (nt 449-487) with B-box (5’-CGGTCGATG-3’) changed to 5’-GCCAGCTAC-3’(GC⇒CG; AT⇒TA);Cm^R^ | [3]; Fig. S4 |
| pTVC190 | pTVC243 + *oriII* (nt 473-487); Cm^R^ | Fig. S4 |
| pTVC210 | Same as pTVC122, but P*rctB* is fused to *lacZ*; Ap^R^ | Figs. 2, S3, S9 |
| pTVC211 | Same as pTVC123, but P*rctB* is fused to *lacZ*; Ap^R^ | Figs. 2, S9 |
| pTVC221 | pTVC243 + *rctA* lacking *parS2-B* (nt 138-246); Cm^R^ | Fig. 4 |
| pTVC222 | pTVC243 + 39-mer (nt 449-487); Cm^R^ | [5,6,7]; Figs. 6, S4 |
| pTVC228 | pTVC243 + *oriII* (nt 788-934); Cm^R^ | Fig. 4 |
| pTVC233 | *parB2* (nt 1071010-1069967) under the constitutive promoter in pPS69; Ap^R^ | Source of *parB2* present in pTVC236 |
| pTVC234 | P*repA* from P1 (nt 556-600) fused to *lacZ* in pMLB1109; Ap^R^ | [3]; Fig. 3 |
| pTVC236 | pACYC184 with Pconst-*parB2* from pTVC233; Cm^R^ | Figs. 2, S2, S3, S9 |
| pTVC239 | pTVC234 + *oriII* (nt 90-299) upstream of P*repA-lacZ*; Ap^R^ | Fig. 3 |
| pTVC243 | pTVC61 derivative with 400 bp shorter transcription-free region (200 bp from each side of MCS); Cm^R^ | [3]; Figs. 4, S4  Used for cloning |
| pTVC248 | pTVC243 + *oriII* (nt 291-445); Cm^R^ | Fig. 4 |
| pTVC270 | pTVC243 + *oriII* (nt 487-717); Cm^R^ | Fig. 4 |
| pTVC284 | Same as pTVC222, but *oriII* fragment contains 11bp-39mer-11bp native sequence (nt 439 – 498) | The source of insert in pTVC371 |
| pTVC291 | pTVC243 + *rctA* (nt 98-246); Cm^R^ | Figs. 4, 6 |
| pTVC330 | pTVC222 with deleted 10 bp from the left part of the IR within the 39-mer; Cm^R^ | [3]; Fig. S4 |
| pTVC332 | pTVC222 with deleted 10 bp from the middle part of the IR within the 39-mer; Cm^R^ | [3]; Fig. S4 |
| pTVC371 | pNEB193 + 39-mer with 100 bp flanks from pTVC284;Ap^R^ | Fig. S5 |
| pTVC400 | pTVC243 + *oriII* (nt 1083 - 1124); Cm^R^ | Fig. 4 |
| pTVC499 | pACYC184 + *rctB* (nt 1118-3115) under P_BAD_ from pTVC11 | Figs. 3, 7, S3 |
| pTVC501 | *parB2* (nt1071010-1069967) under *lacI*^q^-P*lac* in pTVC510;Sp^R^ | Figs. 3, 7, S2, S3, S8, S9 |
| pTVC504 | Same as pTVC239, but *oriII* (nt 90-520); Ap^R^ | Fig. 3 |
| pTVC505 | Same as pTVC239, but *oriII* (nt 90-446); Ap^R^ | Fig. 3 |
| pTVC508 | *parAB2* (nt 1072250-1069967) in pTVC510; Sp^R^ | Fig. S9 |
| pTVC509 | Same as pTVC239, but *oriII* (nt 90-718); Ap^R^ | Fig. 3 |
| pTVC510 | pTVC17 deleted for *rctB* but retains *lacI*^q^-P*lac*; Sp^R^ | Figs. S2, S3 |
| pTVC514 | pTVC243 + *oriII* (nt 707 to 775); Cm^R^ | Fig. 4 |
| pTVC515 | pTVC243 + 39-mer (nt 438 to 520); Cm^R^ | Fig. 4 |
| pTVC520 | pTVC158 + 39-mer from pTVC515; Cm^R^ | Fig. S1*B* |
| pTVC521 | pTVC158 *+parS2-B* from pTVC526; Cm^R^ | Fig. S1*B* |
| pTVC525 | pTVC243 + 39-mer (same as pTVC222 except IR mut.); Cm^R^ | Fig. S4 |
| pTVC526 | pTVC243 + *parS2-B* (nt 108-143); Cm^R^ | Fig. 4 |
| pTVC529 | pTVC234 + 39-mer (nt 438 – 520); Ap^R^ | Fig. S9 |
| pTXB1 | Cloning vector for protein purification; Ap^R^ | NEB |
| pUT18C | Bacterial two-hybrid prey vector; Ap^R^ | Euromedex; Fig. S8 |

*coordinates are from GenBank (Accession Number NC_002506)

**References**

1. Simons RW, Housman F, Kleckner N (1987) Improved single and multicopy *lac*-based cloning vectors for protein and operon fusions. Gene 53: 85-96.

2. Fekete RA, Chattoraj DK (2005) A cis-acting sequence involved in chromosome segregation in *Escherichia coli*. Mol Microbiol 55: 175-183.

3. Venkova-Canova T, Chattoraj DK (2011) Transition from a plasmid to a chromosomal mode of replication entails additional regulators. Proc Natl Acad Sci U S A 108: 6199-6204.

4. Brendler TG, Abeles AL, Reaves LD, Austin SJ (1997) The iteron bases and spacers of the P1 replication origin contain information that specifies the formation of a complex structure involved in initiation. Mol Microbiol 23: 559-567.

5. Jha JK, Baek JH, Venkova-Canova T, Chattoraj DK (2012) Chromosome dynamics in multichromosome bacteria. Biochim Biophys Acta 1819:826-829.

6. Jha JK, Demarre G, Venkova-Canova T, Chattoraj DK (2012) Replication regulation of *Vibrio cholerae* chromosome II involves initiator binding to the origin both as monomer and as dimer. Nucleic Acids Res 40:6026-6038.

7. Venkova-Canova T, Saha A, Chattoraj DK (2012) A 29-mer site regulates transcription of the initiator gene as well as function of the replication origin of *Vibrio cholerae* chromosome II. Plasmid 67: 102-110.

8. Lemonnier M, Bouet JY, Libante V, Lane D (2000) Disruption of the F plasmid partition complex in vivo by partition protein SopA. Mol Microbiol 38: 493-505.

9. Churchward G, Belin D, Nagamine Y (1984) A pSC101-derived plasmid which shows no sequence homology to other commonly used cloning vectors. Gene 31: 165-171.

10. Srivastava P, Demarre G, Karpova TS, McNally J, Chattoraj DK (2007) Changes in nucleoid morphology and origin localization upon inhibition or alteration of the actin homolog, MreB, of *Vibrio cholerae*. J Bacteriol 189: 7450-7463.

11. Pal D, Venkova-Canova T, Srivastava P, Chattoraj DK (2005) Multipartite regulation of *rctB*, the replication initiator gene of *Vibrio cholerae* chromosome II. J Bacteriol 187: 7167-7175.

12. Venkova-Canova T, Srivastava P, Chattoraj DK (2006) Transcriptional inactivation of a regulatory site for replication of *Vibrio cholerae* chromosome II. Proc Natl Acad Sci U S A 103: 12051-12056.
