# Supplementary material for: Evidence for Two Different Regulatory Mechanisms Linking Replication and Segregation of Vibrio cholerae Chromosome II
Source: PLoS Genet. 2013 Jun 20;9(6):e1003579. doi: 10.1371/journal.pgen.1003579 (PMC3688505; doi:10.1371/journal.pgen.1003579)
Supplement: Table S2 — Primers used in this study. (DOCX) [file pgen.1003579.s011.docx]

**Table S2.** Primers used in this study

| **Used for** | **Primer name** | **Sequence (5’ – 3’)** |
| --- | --- | --- |
| CVC2336 | *parS2-B*#1-lg | TGTGGATTCAAGAATAGGCGTAATGAAC |
|  | 1st_par#2 | CTAGAAAGTATAGGAACTTCAAGTGACACTGTAATATTTAAAATGTTTTC |
|  | 2nd_par#2 | TATTCCGAAGTTCCTATTCTCTAGAAAGTATAGGAACTTCAAGTGAC |
|  | 3rd_par#2 | ATCTTGAAGTTCCTATTCCGAAGTTCCTATTCTCTAGAAAG |
|  | *parS2-B*#6-lg | CAATGATCAACGTAAGCTTCTAAGCATG |
|  | par#5-int | TTCTAGAGAATAGGAACTTCCCACGTTTTGAAGTGATGATGAATAAATAAAAG |
|  | 2nd_par#5 | CGCTCTGAAGTTCCTATACTTTCTAGAGAATAGGAACTTCCCAC |
|  | 3rd_par#5 | CCAGCTTCAAAAGCGCTCTGAAGTTCCTATACTTTCTAGAG |
|  | 3rd_par#3 | GAACTTCGGAATAGGAACTTCAAGATCCCCCACGCTGCCG |
|  | 3rd_par#4 | GAACTTCAGAGCGCTTTTGAAGCTGGGGTGGGCGAAGAACTCC |
|  | *parS2-B-*lg_seqUP | CTTGCACCCCATCTGGCACATTATCCAG |
|  | *parS2-B-*lg_seqDW | CAGAAGCATGATCATGTATCGATGGAAACC |
| Fig. S7 *parB2* | BJH179  BJH222 | GGCTTGCATCTCTTTACCT  GGTGTATGTGGCTGAAAA |
| Fig. S7 *parA2* | BJH178  BJH221 | GCTGGATAATGTGCCAGAT  GCTGGATAATGTGCCAGAT |
| Fig. S7 *parS2-B* | TVC50  TVC150 | AGTCATGAATTCGATGAGAAAATGCCTATCTTTC  AGAAAAAGACTCGAGAAATAAAGAAAAATTTGTTGTTTTTAAA |
| Fig. S7 39-mer | TVC59  TVC67 | TCAAGAGGATCCTCGTCGCGGAAGCATGTAAA  GCATGATCTCGAGTGTTGTTCTGTTTATCTTG |
| Fig. S7  12+11 mer | TVC61  TVC65 | TAACAAGGATCCGTCAAAACAAGATAAACAGAAC  TCGTACTCTCGAGACCACGGAAAGAT |
| Fig. S7 P*rct*B | DP18  TVC16 | CCCGAATTCCTGAATGGCTTAAAATAATCTC  TTCTGAGCTCATGGTTAGATCCGTATCACAC |
| Figure S7 *rctB* | BJH310  BJH311 | CCTGTGGAAGGCAGTGAT  CTCCGGTGGAAATAGGAA |
| Fig. S7 *parS1* | BJH45  BJH46 | TTGGAGATGGTTTGTGCATT  AGATAGAGAGCGAATGTTAG |
| pTVC505 | TVC293  TVC305 | CCCACATATTCTTCGATAAGAATTCAACATTTTAAATATTACA  TTTACATGCTTCGAATTCGATTTACCTCTTGATCATC |
| pTVC508 | BJH172  BJH175 | AACTAGCTAGCACAATCTGTTTAAAGCCCTA   TAAGGCTCGAGCCCCTAAAACGCACAAAGCC |
| pTVC509 | TVC293  TVC307 | CCCACATATTCTTCGATAAGAATTCAACATTTTAAATATTACA  TCGTACTGAATTCACCACGGAAAGATGATCAAGAGA |
| pTVC514 | TVC513  TVC514 | GATCCTTTCCGTGGTCATGAGAGTACGAGAGGAAGGAGATACAACGACCTATCTTGATGTCATCCACTCAGGTC  TCGAGACCTGAGTGGATGACATCAAGATAGGTCGTTGTATCTCCTTCCTCTCGTACTCTCATGACCACGGAAAG |
| pTVC515 | TVC515  TVC516 | GATCCGTAAATCGTCGCGGAAGCATGTAAATTCATTATCAATTTACGGTCGATGTCAGGCAGAGTAAGGCTTTGGCTAGTCAGTGATC  TCGAGATCACTGACTAGCCAAAGCCTTACTCTGCCTGACATCGACCGTAAATTGATAATGAATTTACATGCTTCCGCGACGATTTACG |
| pTVC525 | TVC544  TVC545 | GATCCCGGAAGCATGTAAATTCATTATCTTAAATCGGTCGATGC  TCGAGCATCGACCGATTTAAGATAATGAATTTACATGCTTCCGG |
| pTVC526 | TVC546  TVC547 | GATCCAGTGTCACTTATTTACAATGTAAAGCCACGTTTTGAC  TCGAGTCAAAACGTGGCTTTACATTGTAAATAAGTGACACTG |
| pTVC529 | TVC548  TVC549 | AATTCGTAAATCGTCGCGGAAGCATGTAAATTCATTATCAATTTACGGTCGATGTCAGGCAGAGTAAGGCTTTGGCTAGTCAGTGATG  AATTCATCACTGACTAGCCAAAGCCTTACTCTGCCTGACATCGACCGTAAATTGATAATGAATTTACATGCTTCCGCGACGATTTACG |
| pBJH217 | BJH472  BJH473 | GCCACGTTTTGAAGTGCATATGAATAAATAAAAGCGAGCCGTAAGCG  CGCTTACGGCTCGCTTTTATTTATTCATATGCACTTCAAAACGTGGC |
| pBJH218 | BJH475  BJH476 | GGCTTCATATGGGCTTAGAAGTGTATCGCG  GGCTTCATATGCCCGGGGATCCTCGAGTCGA |
