# Supplementary material for: Evidence for Two Different Regulatory Mechanisms Linking Replication and Segregation of Vibrio cholerae Chromosome II
Source: PLoS Genet. 2013 Jun 20;9(6):e1003579. doi: 10.1371/journal.pgen.1003579 (PMC3688505; doi:10.1371/journal.pgen.1003579)
Supplement: Text S1 — Supporting Materials and Methods. (DOC) [file pgen.1003579.s012.doc]

**Text S1** Supporting materials and methods

Cloning of *parB2* and *parA2B2*

To construct inducible sources of *parB2* and *parA2B2*, the genes were first amplified by PCR (using oligonucleotides BJH174 and BJH175 for *parB2*, and BJH172 and BJH175 for *parA2B2*, Table S2) from total DNA extracted from *V. cholerae* (CVC209). The fragments were ligated to the vector backbone of pTVC17, downstream of *lacI^q^*-P*lac*, between unique NheI and XhoI sites. The vector backbone of pTVC17 (pSC101*ori*) was obtained after removal of the *rctB-luciferase* fusion with same ends. The resulting plasmids were pTVC501 (*parB2*) and pTVC508 (*parA2B2*). Vector control (pTVC510) was obtained by simple blunting and subsequent re-ligation of the pTVC17 NheI-XhoI vector backbone.

To construct a constitutive source of *parB2*, a PCR product carrying the gene (amplified using oligonucleotides TVC300 and TVC301) was first ligated to a vector backbone of pPS69 between XhoI and SalI sites. The cloning resulted in placing *parB2* downstream of the constitutive P*trc* promoter [1]. The new plasmid was called pTVC233. The P*trc-parB2* fragment was recloned into a lower copy number vector with a different drug resistance marker (pACYC184, Cm). Plasmid pTVC233 was digested by NsiI, and SalI, treated by DNA Polymerase I (NEB) and the fragment containing P*trc-parB2* was transferred to pACYC184 after linearizing with EcoRV. This cloning resulted in pTVC236.

Cloning of *rctB*

To avoid incompatibility and similar antibiotic-resistance, HindIII-digested and Klenow-treated fragment carrying *araC*-P*bad*-*rctB* fusion from pTVC11 was transferred to pACYC184 that was linearized by EcoRV and de-phosphorylated by SAP. The resulting plasmid was called pTVC499.

Plasmid stability assay

A two-plasmid system was used to test the centromeric function of the central 39-mer: one plasmid was miniF (pDAG203 or its derivatives, Cm^R^) and the other contained *parAB2* under the control of an IPTG-inducible promoter (pTVC508, Sp^R^). miniF derivatives either had the *parS2-B* site (as in pBJH180) or the central 39-mer (as in pBJH181). DH5Δ*lac* cells carrying both miniF (Cm^R^) and *parAB2* (Sp^R^) plasmids were grown in minimal medium [M63 buffer, glucose (0.4%), casamino acids (0.2%), CaCl_2_ (0.05 mM), MgSO_4_ (5 mM) and vitamin B1 (1 μg/ml)] from single colonies that were grown under selection of both the plasmids using 40 μg/ml spectinomycin and 25 μg/ml chloramphenicol. The cultures were subsequently grown in the presence of spectinomycin only and diluted several times to maintain them in logarithmic phase. At different intervals, the culture was monitored for OD_600_ and plated on LB plates containing spectinomycin. After overnight incubation the colonies were stabbed onto spectinomycin+chloramphenicol plates to assess miniF plasmid retention. 120 colonies were stabbed for each point of Fig. S6. The number of cell generations was deduced from OD_600_ values and the dilution factors.

Western Blotting

Cultures of plasmid carrying cells were grown to middle log phase (OD=0.4-0.5) and a total OD_600_ = 2 and 1 equivalent of *V. cholerae* CVC209 and *E. coli* (BR8706 carrying plasmids) cells, respectively, were harvested by centrifugation for 5 min at 13,000 rpm using a microfuge (Sorvall, Rotor 3325B) at RT. The pellets were stored frozen. To assay for proteins, each of the pellets was thawed and resuspended in 100 μl 1x SDS Red Loading Sample Buffer with 1x DTT (NEB, Beverly, MA), vortexed, boiled for 7 min, vortexed again and finally spun for 5 min 13,000 rpm at RT in a microfuge. Different aliquots from the supernatant were loaded on either 7.5% or 10 % Tris-HCl Pre-cast Criterion gel (Bio-Rad, Hercules, CA). The gel was run in 1x Tris-HCl SDS buffer (Bio-Rad, Hercules, CA) at constant 200V for ~45-50 min, until the front dye reached the gel bottom. The gel was washed in distilled water and the proteins were transferred to the membrane of the ready-made “sandwiches” (Invitrogen, Carlsbad, CA) using *iBlot Fast Transfer* apparatus according to the manufacturer’s protocol (Program P2; Invitrogen, Carlsbad, CA). The transfer was nearly 100% completed in 7 min. The membrane was blocked for 1 hour in 5% blocking reagent (GE Healthcare, Piscataway, NJ) in PBS-T (1x PBS from KD Medical, Columbia, MD, with 0.1% Tween 20 from Affymetrix, Santa Clara, CA). Next, the membrane was incubated with purified ParB2 antibody (1:7,500 dilution in 5% blocking reagent in PBS-T) for 1 hour. The membrane was washed with PBS-T three times, 20 min each time. Next, the membrane was incubated for 1 hour in 5% blocking reagent in PBS-T containing a 1:5,000 dilution of anti-rabbit horseradish peroxidase-conjugated secondary antibody from donkey (GE Healthcare, Piscataway, NJ). The membrane was finally washed three times as before and kept in fresh PBS-T before staining and imaging. Four ml of each detection solutions of SuperSignal* West Pico Chemiluminescent ECL Substrate (Thermo Scientific, Waltham, MA) were mixed and incubated with the membrane for 1 min. The images were visualized using Fuji LAS-3000 camera. The intensities of the bands were evaluated using Image Guage 4.1 (FujiFilm).

DNase I footprinting Assay

One microgram of PAGE-purified oligonucleotides TVC256 (5'-AATTCGAGCTCGGTACCC -3') and TVC257 (5'-AGCTTGCATGCCTGCAGG -3') were end-labeled using 30 units T4 Polynucleotide Kinase (NEB) and 50µCi of [γ^32^-ATP] (Perkin-Elmer), and cleaned of free label by passage through Mini Quick Spin Oligo Columns G-25 (Roche diagnostics). The oligonucleotides (50 ng each) were used as primers to separately label the two strands of a 39-mer-containing fragment by PCR, using unlabeled complementary strand primers and Phusion Polymerase (NEB). The template was 1 ng of phenol-purified mini-prep of pTVC371 DNA (Qiagen). The single-end labeled PCR products carried the 39-mer with 11 bp natural flanks of *oriII* sequences followed by 148 bp and 154 bp of vector sequences at the ends. The PCR products were purified through Min-Elute Qiagen columns. The radioactivity of the fragments used in EMSA was about 25,000 cpm. The binding reactions were treated with 0.01 units of DNase I (Promega) for 1 min at 30°C, and loaded immediately onto a 5% PAGE. After a 2 h run at a constant 175V, the gel was exposed overnight to Kodak film. The bound and unbound species were gel-extracted by crush and soak, followed by phenol-purification and ethanol-precipitation. The DNA was desalted by passing through mini Quick Spin DNA Columns G-25 (Roche) equilibrated in water. The DNA was dried under vacuum and resuspended in different volumes of water to achieve nearly equal cpm/µl for each sample. Equal volumes of DNA were mixed with formamide-containing stop solution supplied with the fmol cycle sequencing kit (Promega). The same kit was used to obtain the size markers using plasmid pTVC371 as a template and the labeled oligonucleotides used above to make the labeled fragments. One thousand cpm was loaded per lane of a 6% sequencing gel (SequaGel 6, National Diagnostics), after heating for 5 min at 90°C, along with the sequencing reactions. The gel was run using the SA-43 apparatus **(**Life Technologies) for 2 h at constant 40 W in 1x TBE buffer. The gel was dried for 1 hour at 80°C and exposed to a Fuji phosphorimager plate for 48 hours. The band intensities were recorded using the FLA-5100 phosphorimager (Fujifilm, Edison, NJ).

Construction of Δ*parS2-B*::FRT strain CVC2336

The strain was created by exchanging the *parS2-B* site (15 bp) for the FRT site (34 bp) [2]. The strain manipulations were done as described [3]] with minor modifications. Briefly, plasmid pBR-flp-hapR*, which apart from the FLP recombinase gene contains a functional *hapR* allele, was introduced into N16961 (CVC2335) by electroporation. This allows the resultant strain to become naturally competent upon growth on chitin surfaces. Chitin flakes were used as an inducer of natural competence and a PCR-derived DNA fragment was used as transforming DNA [4]. The PCR fragment was obtained by several rounds of PCR to gradually include one FRT site to each of the amplified *parS2-B* flanking regions. The upstream flanking region of *parS2-B* was amplified using genomic DNA of N16961 as template and primers parS2B#1-lg & 1st_par#2 (1^st^ PCR) / 2nd_par#2 (2^nd^ PCR; 1^st^ PCR fragment as template) / 3rd_par#2 (3^rd^ PCR; 2^nd^ PCR fragment as template). Likewise, the downstream flanking region of parS2-B was amplified using the oligonucleotides parS2B#6-lg & par#5-int (1^st^ PCR) / 2nd_par#5 (2^nd^ PCR; 1^st^ PCR fragment as template) / 3rd_par#5 (3^rd^ PCR; 2^nd^ PCR fragment as template). A kanamycin resistance cassette (*aph*) was amplified using primers 3rd_par#3 & 3rd_par#4 and plasmid pBR-FRT-Kan-FRT as template. Due to their overlapping ends, the three resulting PCR fragments (upstream and downstream region and the *aph* containing fragment) were then used as template in a final PCR reaction in which parS2B#1-lg and parS2B#6-lg served as oligonucleotides (final length of PCR fragment was 2825 bp). For all PCR reaction a high-fidelity polymerase (Pwo DNA polymerase from Roche) was utilized to avoid undesired point mutations. After the PCR fragment was taken up by the naturally competent *V. cholerae* N16961 / pBR-flp-hapR* cells, transformants were selected based on the two antibiotic resistance genes encoded by the integrated PCR fragment containing *aph* and plasmid pBR-flp-hapR* carrying *bla*, respectively. Correct insertion of (parts) of the PCR fragment resulting in deletion of the *parS2-B* site was confirmed by colony PCR. The *aph* cassette, which was located between the two PCR-generated FRT sites, was excised through induction of the *flp* gene (pBR-flp-hapR*) by a temperature-upshift [3]. Finally, plasmid pBR-flp-hapR* was cured by growth in rich medium [3]. The final strain, N16961Δ*parS2-B*::*FRT*, was verified by diverse PCR reactions for the exchange of the *parS2-B* site by one FRT site. The whole modified region of interest (1031 bp) was sequenced after PCR amplification with primers parS2B-lg_seqUP and parS2B-lg_seqDW. The FRT site was found to have a A3C change.

Fluorescence microscopy

Cells were grown in LB at 37°C to saturation and further concentrated by centrifugation and resuspension in the same medium. The cells were imaged using a Nikon (E1000) microscope fitted with a Hamamatsu EM-CCD digital camera (Photonics KK) and controlled by the MetaMorph Premier software program (Molecular Devices, Sunnyvale, CA), as described [5].

**References**

1. Srivastava P, Demarre G, Karpova TS, McNally J, Chattoraj DK (2007) Changes in nucleoid morphology and origin localization upon inhibition or alteration of the actin homolog, MreB, of *Vibrio cholerae*. J Bacteriol 189: 7450-7463.

2. Yamaichi Y, Fogel MA, McLeod SM, Hui MP, Waldor MK (2007) Distinct centromere-like *parS* sites on the two chromosomes of Vibrio spp. J Bacteriol 189: 5314-5324.

3. Silva Ode S, Blokesch M (2010) Genetic manipulation of *Vibrio cholerae* by combining natural transformation with FLP recombination. Plasmid 64: 186-195.

4. Marvig RL, Blokesch M (2010) Natural transformation of *Vibrio cholerae* as a tool--optimizing the procedure. BMC Microbiol 10: 155.

5. Kadoya R, Chattoraj DK (2012) Insensitivity of Chromosome I and the Cell Cycle to Blockage of Replication and Segregation of *Vibrio cholerae* Chromosome II. MBio 3: 00067-00012.
